# Insects in bioregenerative life support systems: unlocking their role in space sustainability

**DOI:** 10.3389/fphys.2025.1621099

**Published:** 2025-09-10

**Authors:** Åsa Berggren, Annette Bruun Jensen, David Copplestone, Roberto Guidetti, Martina Heer, Paola Pittia

**Affiliations:** ^1^ Department of Ecology, Swedish University of Agricultural Sciences, Uppsala, Sweden; ^2^ Department of Plant and Environmental Sciences, University of Copenhagen, Frederiksberg, Denmark; ^3^ Biological and Environmental Sciences, University of Stirling, Stirling, United Kingdom; ^4^ Department of Life Sciences, University of Modena and Reggio Emilia, Modena, Italy; ^5^ IU International University of Applied Sciences, Bad Reichenhall, Germany; ^6^ Institute for Nutritional and Food Sciences, University of Bonn, Bonn, Germany; ^7^ Department of Bioscience and Technology for Food Agriculture and Environment, University of Teramo, Teramo, Italy

**Keywords:** bioregenerative life support systems, edible insects, space food production, nutrient cycling, circular systems

## Abstract

Long-duration space missions and planetary colonization efforts will depend on Bioregenerative Life Support Systems (BLSS) for sustainable food production, water recycling, and waste management. However, most BLSS research to date has focused almost exclusively on plants, with limited attention to animals and species-level ecological interactions. Here, we review 280 BLSS-focused studies and identify significant underrepresentation of insects and invertebrates, despite their multifunctional potential for nutrient recycling, protein production, and ecological resilience. Only 13 studies experimentally included insects, and these are rarely explored in interactions with other species in the system. Insects such as *Acheta domesticus*, *Tenebrio molitor* and *Bombyx mori* show promise but remain underexamined under space-relevant conditions. Comparisons with terrestrial circular food systems reveal parallel knowledge gaps but also highlight emerging evidence supporting invertebrates as integral components. We argue that closing these gaps will require targeted research on insect physiology and species interactions under space-like stressors such as microgravity and radiation. Drawing on insights from Earth-based circular food systems can accelerate the integration of multifunctional insect species into closed-loop space habitats. Addressing these gaps is essential to create robust, resilient bioregenerative systems that can support human life beyond Earth.

## 1 Introduction

The idea of Bioregenerative Life Support Systems (BLSS) or Closed Ecological Life Support Systems (CELSS) is to generate vital resources that humans need for survival in places other than on Earth. While both CELSS and BLSS aim to sustain life by recycling air, water, and waste and by producing food, they differ slightly in emphasis. CELSS refers to a technologically controlled system mainly focusing on crop production and resource management. In contrast, BLSS adopts a broader, ecosystem-based approach, integrating diverse biological components into a self-regulating regenerative system (Liu et al., 2021). The obvious interest in BLSS/CELSS (hereafter “BLSS”) for crewed space travel began in the 1950s, and since then varying levels of research have been allocated to examine their viability in this context (Mason and Carden, 1982, Munns and Nickelsen). They are generally regarded as techno-ecological systems that both utilise and generate biological resources, replicating Earth-like functions within a closed-loop framework requiring minimal external input once established. However, replicating Earth’s biotic and abiotic factors is a formidable challenge, partly because we still lack a full understanding of how many organisms function and interact in their natural environments (Mkenda et al., 2019; Mengist et al., 2020). Thus, despite the desire for BLSS development, the complexity of ecological interactions has hampered their development.

Successfully implementing BLSS strongly relates to how long such a system must function, and exactly what functions it needs to provide. However, identifying these parameters has changed over time, with assumptions and specifications varying according to political visions and decisions (Munns and Nickelsen 2021). What remains clear is that if humans are to undertake long-duration space missions and planetary colonisation (e.g., Moon or Mars), development of BLSS to replicate various biological processes is necessary (Verseux et al., 2022). While gas, water and waste recycling require regulation by such systems, one of the most challenging and necessary components is the production of a suitable quantity of nutritious food.

The idea of creating a system that provides humans with food in a space environment has been referred to as “space agriculture,” suggesting that the transference of food production to a non-terrestrial environment will likely utilise knowledge used in the practise of agriculture on Earth (Wheeler et al., 2023). Terrestrial agriculture depends not only on food production practices but also on a range of supporting and regulating ecosystem services from the ecosystems they are contained within (Power, 2010). For example, composting materials by soil macro- and microorganisms, pollination of flowers by bees and hoverflies, and predation of plant pests by predatory insects and birds. Thus, to make space agriculture viable for more than a short-term cycle, vital ecosystem services like these will need to be identified and included or replicated in some way. In practice, BLSS will be simplified systems compared to the complex Earth-based ecosystems they are aiming to replicate and hence will contain fewer supporting and regulating components that require implementation. However, this simplification creates its own problems through reduced redundancy in the system (Oliver et al., 2015). Most of Earth’s ecosystems are incredibly complex, with ecosystem functional traits often replicated in more than one species (Luza et al., 2023). This means that if one component of the system is removed or fails, other species with similar functions can largely replace them, creating system stability. With very simple ecosystems, like BLSS, the components will have little “backup” capability, meaning that each component’s functions and interactions will need to be very well understood to ensure ongoing effective cycling of gas, water, waste and food production. Therefore, a comprehensive understanding of the ecology of species being considered for BLSS inclusion is essential for their long-term success. Given that many species and ecological processes remain poorly understood (Mkenda et al., 2019; Mengist et al., 2020), this gap in terrestrial ecological knowledge likely represents the greatest challenge to developing effective BLSS.

On Earth growing insects for mass consumption is rapidly increasing (Berggren et al., 2019; Siddiqui et al., 2023), with four insect species, including House crickets (*Acheta domesticus*) and Yellow mealworms (*Tenebrio molitor*), approved for human consumption by the European Commission (European Commission, 2023). The main reasons for the current interest in insect mass-rearing are the insects’ nutritional values (Rumpold and Schlüter, 2013; Makkar et al., 2014; Jantzen da Silva et al., 2020), and being sources of essential nutrients (in particular, amino- and fatty acids) (Hermans, 2021; Magara, 2021; Skotnicka et al., 2021). Several species also utilise different feed resources efficiency and can convert organic matter and water into protein (Nakagaki and DeFoliart, 1991). This conversion efficiency has major implications for creating sustainable and circular food production systems with insects as an integral part (Berggren et al., 2019).

In this review, we examine the published scientific literature on BLSS to help gain an understanding of state of knowledge of food production in the context of future space exploration. By comparing BLSS-specific knowledge with the ongoing parallel development of circular food systems on Earth and the taxa and traits highlighted there, our aim is to identify current knowledge gaps in order to highlight future research priorities and ways forward in the development of food production in BLSS. We put an emphasis on animals, and particularly the acknowledged roles of insects in these systems. Broader physiological and technical questions related to organismal physiology and food production under space conditions lie outside the scope of this paper.

## 2 Methods

We searched the Web of Science database for English-language papers published up to and including 2024 using the topic terms “Bioregenerative Life Support Systems,” “BLSS,” “Closed Ecological Life Support Systems,” or “CELSS.” This resulted in 1 812 papers. From these, we examined the accessible metadata (title, keywords, and abstract) of each paper and removed papers whose primary focus was not BLSS, CELSS or acronyms and words with this same meaning (e.g., bioregenerative systems). We also excluded papers centred on human health, physiology, or behaviour, as our focus was on the systems themselves rather than their effects on humans. This resulted in 435 papers remaining. Of these, we were able to access 280 of the papers for analyses (Supplementary Table S1). From these papers, we extracted data on the study type (theoretical/modelling, review, or experimental), primary focus, and whether plants or animals were used, including the species involved. We also recorded whether studies examined species interactions and, if so, which species were involved. As the papers included in the study are all in English, we note that our work does not include studies published in other languages from researchers that might also be working on BLSS.

## 3 Published research on food production in the BLSS

**FIGURE 1:**
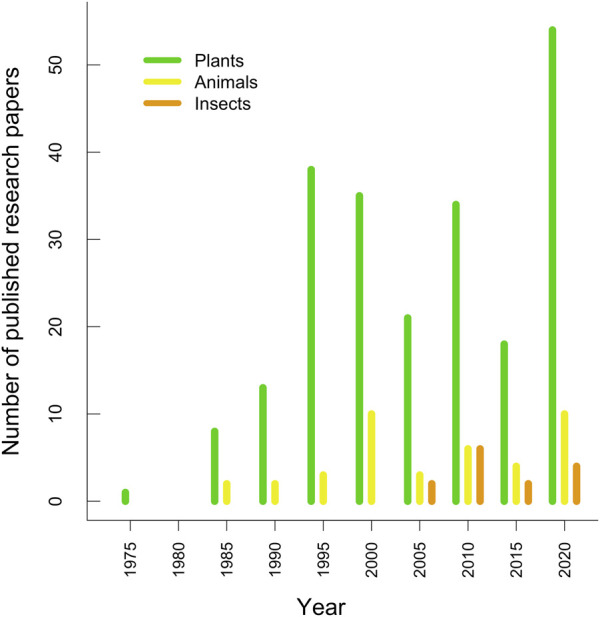
Number of scientific papers on food production in Bioregenerative Life Support Systems (BLSS) from 1975–2024, where the main focus has been on plants, animals and insects. The x-axis shows 5-year summaries starting from the labelled year (e.g., 1975 is from 1975–1979; 2000 is from 2000–2004).

The number of BLSS-focused publications has grown since the first recorded paper in 1978, reflecting an interest in sustainable life support. A trend emerges, where plant studies dominate the literature consistently, indicating a prioritisation of primary producers in closed-loop system research. In contrast, research on animal integration has been limited, with only about one animal-focused paper published annually compared to an average of 4.7 plant-related papers per year (Figure 1). This disparity highlights a potential research bias and suggests a significant knowledge gap regarding heterotrophic organisms in BLSS design.

### 3.1 Plants in the system

**FIGURE 2:**
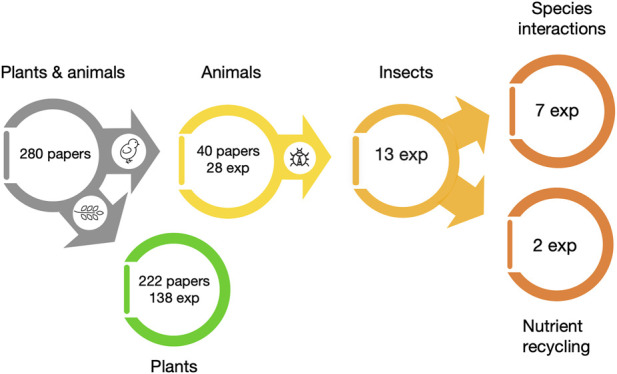
Numbers and proportions of scientific papers published on plants, animals and insects in food production within Bioregenerative Life Support System (BLSS). Data also show the number of papers that were experimental studies (exp) and their focus for two important features for BLSS (waste recycling and species interactions).

Of the 280 papers analysed, 222 (79%) addressed plant production in some capacity (Figure 2), underscoring the central role of autotrophs in bioregenerative systems. Among these, 84 were modelling-based or theoretical, reflecting ongoing efforts to simulate and optimise plant growth in controlled environments. The remaining 138 experimental studies predominantly investigated fundamental aspects such as plant growth (23.9%) and photosynthesis (19.6%). Other areas such as light spectrum responses (8.7%), biomass production (5.8%), gravitropism (4.3%), and nutrient uptake and recycling (2.9%) received comparatively less attention, although they are critical for system resilience in space-like conditions. Notably, a wide variety of plant species and varieties have been tested, but lettuce (*Lactuca sativa*) and wheat (*Triticum aestivum*) emerge as the most commonly studied species. However, only a fraction of these studies was conducted under actual or simulated space conditions (e.g., microgravity or altered atmospheric composition) (e.g., Yang et al., 2019; Sathasivam et al., 2021). The heavy reliance on Earth-based findings may limit our understanding of plant performance in space and underrepresents the systemic challenges that future BLSS implementations may face. While photosynthesis and biomass production are foundational processes, the relative underrepresentation of studies examining plant-soil-microbe interactions, secondary metabolites, and stress responses may hinder comprehensive BLSS development. These areas are vital to plant health, nutrient efficiency, and food quality, parameters that become even more critical in the confined, resource-limited conditions of space habitats.

### 3.2 Animals in the system

Only 40 papers addressed animal integration within BLSS, with 28 of these studies (70%) comprising experimental work (Figure 2). The remaining papers were either modelling studies or reviews. The experimental studies encompassed both terrestrial and aquatic species, such as the freshwater snail (*Biomphalaria glabrata*), Tilapia fish (*Oreochromis spp*.), quail (*Coturnix coturnix*) and earthworm (*Eisenia fetida*) (e.g., Gonzales et al., 2009; Romano et al., 2023). Insects appeared in only 13 of the animal studies (Figure 2, Table 1). The silkworm (*Bombyx mori*) was the most studied insect species (eight studies), followed by the yellow mealworm (*T*. *molitor*) (two studies), the black soldier fly (*H*. *illucens*) (two studies) and a mix of different species in one study. Notably, the biological factors examined were similar to those used in plant studies; individual growth and survival, but also animal specific features like development and metamorphosis and nutrient assimilation.

**TABLE 1:** Summary of insect-based studies relevant to bioregenerative life support systems (BLSS). The table outlines the insect species examined, the focus of each study, major findings, and study-specific recommendations. Studies span nutritional assessments, waste conversion, gas exchange roles, microbial interactions and system integration.

Focus species	Examined	Main results	Recommendations	Publication data
*Bombyx mori* (silkworm)	Effect of lettuce feeding in BLSS on gut microbiota diversity	Silkworms fed with lettuce had reduced gut bacterial diversity and physiological activity	Consider using dominant beneficial gut bacteria as probiotics to improve nutrient absorption and health in BLSS	Liang et al. (2014)
*Bombyx mori* (silkworm)	Respiration characteristics and nutritional composition of silkworms under BLSS feeding	Silkworms had different respiration rates when fed mulberry vs. lettuce; silkworm powder is nutritionally rich with high protein, vitamins, and fatty acids	Use silkworms in BLSS for efficient protein production using inedible plant biomass	Tong et al. (2011)
*Bombyx mori* (silkworm)	Growth and protein yield from silkworms fed artificial diet vs. lettuce leaves for space protein provisioning	Silkworms grew well on lettuce leaves, achieving ∼70% protein content. Biotransformation rate over 70%. 2.2 m³ of space could feed 7 astronauts daily.	Silkworms are feasible protein sources in BLSS. Lettuce leaves can be effective feed. Culture conditions must be optimised for space use.	Yang et al. (2010)
*Bombyx mori* (silkworm)	Nutritional value, feeding method, environmental impact, and protein processing from silk for space diets	Silkworms and silk are high in protein; edible protein can be derived from silk. Silkworms are compact, efficient, and nutrient-dense.	Silkworms including silk are suitable protein sources for BLSS. Processing methods for edible silk protein should be developed.	Yang et al. (2009)
*Bombyx mori* (silkworm)	Growth comparison of silkworms in and out of a closed multibiological life support system (BLSS)	No significant differences in biomass or growth indicators between BLSS and external environment. Silkworms showed slower growth in BLSS.	Silkworms can be effectively cultivated in BLSS. Models for silkworms can support system optimisation.	Tong et al. (2012)
*Bombyx mori* (silkworm)	Gut bacterial communities and enzyme-producing bacteria (cellulase, amylase) in silkworms reared in BLSS vs. traditional conditions	Bacterial diversity decreased in BLSS; different dominant enzyme-producing microbes identified in BLSS compared to traditional.	Probiotic development using dominant gut bacteria (e.g., Enterococcus) to enhance nutrient absorption and health of silkworms in BLSS.	Liang et al. (2015)
*Bombyx mori* (silkworm)	Feeding scenario using mulberry and lettuce leaves, and nutritional content of silkworms in BLSS	Silkworms can be partially fed lettuce without significant nutritional loss. Powder from fifth instar larvae had 71.6% protein and high essential amino acid content. 105 silkworms can meet daily protein needs for one person.	Silkworms are suitable for BLSS protein production. Feeding strategy using lettuce helps minimise waste. R-criterion can guide optimization of rearing facilities.	Yu et al. (2008)
*Bombyx mori* (silkworm)	Closed-loop control of gas concentrations in BLSS using microalgae-based dynamic response and simulation	Closed-loop control using microalgae effectively stabilised O2/CO2 concentrations via feedback from light and aeration rate adjustments.	Microalgae are suitable for dynamic gas regulation in BLSS. Real-time feedback systems improve BLSS stability and safety.	Hu et al. (2012)
*Tenebrio molitor* (yellow mealworm)	Feeding feasibility on plant waste (inedible wheat and vegetable biomass) in BLSS	Mealworms fed on fermented plant waste showed good growth (up to 56.15% of control fresh weight), high protein (76.14% DW), and degraded 45.74% lignocellulose.	*T. molitor* is a viable BLSS candidate; plant waste feeding can enhance system closure but frass needs treatment before use as substrate.	Li et al. (2013)
*Tenebrio molitor* (yellow mealworm)	Effects of dietary fibre on larval growth, development, and respiration in BLSS context	Optimal fibre content was found to be 5–10%; 5% promotes early larval growth and successful pupation	Control feed fibre content within 5–10%, with 5% as advisable for palatability and growth	Li et al. (2016)
*Hermetia illucens* (black soldier fly)	Survival and pupation in different substrates including lunar regolith simulant	All larvae pupated, but substrate affected pupation timing and adult emergence. Regolith supported successful development.	*H. illucens* is highly adaptable and suitable for BLSS, including lunar missions. Regolith can serve as a viable pupation substrate	Romano et al. (2024)
*Hermetia illucens* (black soldier fly)	Design and validation of a modular unit for studying bioprocesses in BLSS, including BSF rearing and microgreen cultivation	BSF effectively degrades organic waste in a modular rearing unit; the system supports integration into closed-loop space life support	Use modular, upgradable systems like the GRM for preliminary testing of bioprocesses involving insects in BLSS	Metelli et al. (2023)
Insects (no single species focus)	Food systems for space missions; development of bioregenerative food systems for Mars missions	NASA is shifting from prepackaged food systems to bioregenerative food systems for Mars missions; discussed technical, safety, and quality challenges	Develop bioregenerative systems using crop-based and possibly insect-based production, improve food quality and shelf life, integrate food systems into overall mission planning	Perchonok et al. (2012)

For example, several silkworm studies explored the effects of different feedstocks (lettuce vs. mulberry), showing high protein yields (∼70%) and adaptability to BLSS conditions, albeit with altered gut microbiota and slower growth under closed environments (Yang et al., 2010; Tong et al., 2012; Liang et al., 2014). Some also highlighted the silkworm’s potential as a compact, protein-rich food source with nutritional benefits even from silk derivatives (Yang et al., 2009). *T. molitor* showed promise in degrading lignocellulosic plant waste and producing high-protein biomass when fed optimal fibre levels, making it suitable for biotransformation roles (Li et al., 2013; Li et al., 2016). *H. illucens* demonstrated both survivability in novel substrates such as lunar regolith simulants and efficiency in organic waste degradation using modular rearing units, highlighting its utility for closed-loop nutrient cycling (Romano et al., 2024; Metelli et al., 2023). Two studies explicitly examined the role of animals in waste transformation where waste reduction was measured (Li et al., 2013; Metelli et al., 2023; Figure 2). This low number of studies is surprising given the crucial role of heterotrophs in nutrient cycling. This points to a disconnect between the theoretical understanding of animal utility in BLSS and its practical exploration. Furthermore, few studies evaluated animals in combination with other BLSS components, such as microbial communities or plant species (other than food), limiting insights and our understanding of trophic interactions and system interdependencies that are vital for system stability.

### 3.3 Interactions between species in the system

Research into species interactions, particularly involving animals, was markedly sparse. Among the few studies that exist, aquatic systems predominate. The studies primarily focused on nutrient recycling interactions between fish, plants and microbial communities (e.g., Bluem and Paris, 2001; König et al., 2018; Gonzales, 2009). However, terrestrial species interactions were far less explored, with only seven studies were identified, all involving insects (Figure 2). These primarily identified pairwise interactions of insects and their feed, such as silkworms (*B. mori*) feeding on lettuce (*L. sativa*) or insects and their associated microbial communities (*B. mori* and internal microbes) (Tong et al., 2012; Yu et al., 2008; Liang et al., 2014; Liang et al., 2015). While these studies offer a foundation, they do not yet form a cohesive understanding of ecosystem-level dynamics or redundancy - key features required for system resilience in space. The near absence of multi-trophic interaction studies reflects a critical gap in BLSS research. Effective ecosystem engineering for space requires integrated understanding of species roles, functional redundancy, and ecological synergies. Without this, the systems are vulnerable at losses of a single species.

### 3.4 Knowledge produced over time

The dominance of plant-focused studies in this field, has likely advanced our understanding of how to incorporate plants into BLSS has advanced a fair way. The low number of animal-focused publications, and particularly on insects, indicates that knowledge development in this area has been both limited and slow. This imbalance poses practical challenges, given that plants alone cannot fulfil all the necessary functions in a sustainable life support system. As circular food systems need different components to make them work (producing food, transforming waste and byproducts into resources), plants alone cannot sustain these systems. The potential of promising taxa that supply nutrients to both humans and plants, while also recycling waste, warrants further exploration for the development of BLSS. With the construction of circular food systems, the ecological complexities in species interactions and resource use will become increasingly in focus. This will call for research within these areas and utilisation of current knowledge gathered from similar systems on Earth. Animals, especially insects, offer critical services such as waste conversion, protein synthesis, and nutrient recycling. However, their incorporation into BLSS is impeded by a lack of empirical data. The ecological complexity of circular food systems, which are increasingly emphasised for their sustainability on Earth, will likely be mirrored in space-based systems. As these systems evolve, understanding interspecies dependencies, resource flow, and system-level efficiencies becomes essential. To this end, translating insights from Earth-based circular food systems, where invertebrate integration is gaining traction, could provide a blueprint for accelerating BLSS innovation. These systems can serve as testbeds for hypothesis-driven experiments focused on ecosystem services and resilience under controlled conditions.

## 4 Sustainability of circular systems on Earth and in space

### 4.1 Using Earth-based circular systems to inform BLSS

Studies on circular food systems on Earth has been discussed for some time, stemming from an increased need to develop sustainable food production systems (Kennedy et al., 2020; Toplicean and Datcu, 2024). With a global human population expected to increase to 9 billion by 2050, agricultural practises need to change to sustain the ecosystems and their services that support food production (Rockström et al., 2017). One keyway to achieve sustainable terrestrial food production systems is to incorporate the same principles as for BLSS; i.e., a circular model where resources are utilised, re-utilised and not wasted (Sandström and Kummu, 2023). Similar to BLSS studies, the majority of studies being carried out so far are theoretical in nature and even when circularity is the aim most studies focus on single parts of the system (Sandström and Kummu, 2023). As with BLSS, only a very small proportion of studies examine ecological perspectives on a species level, but in contrast to BLSS studies, using animals within these systems is gaining interest (Van et al., 2019). The potential of insects and other invertebrates as vital parts of circular systems are highlighted in several studies (Cadinu et al., 2020; Derler et al., 2021), and promising experiments have been carried out (Cappellozza et al., 2019).

Creating viable BLSS and circular food systems for sustainable food production on Earth are important to be able to achieve the global strategic goals on sustainable development (United Nations, 2015) as well as for future long-term space travels and planetary colonisations (ESA, 2021; 2022). Utilising knowledge and experiences gathered from different types of systems (aimed towards space and Earth use) will likely lead to a faster and more effective development of both uses (Sreeharsha and Mohan, 2021). One promising example of such cross-sectoral learning is the Mission to Mars initiative, which demonstrates how space-based circularity concepts and horticultural expertise can inform each other to accelerate innovation in sustainable food production for both Earth and space (Vermeulen et al., 2020). What is apparent for both BLSS and Earth-focussed circular systems is that both currently lack the necessary experiments that trial animal species as an integrated source of food, and the associated species’ interactions that will be crucial for long-term system functioning within these simplified ecosystems.

### 4.2 Utilising insects’ capacities in BLSS

Current work in Earth-focused circular systems has highlighted the high potential of invertebrates, especially insects, as key components for waste conversion to food protein (Hamam et al., 2024). This is an area where BLSS food production research would greatly benefit from increased attention. Insects offer a uniquely advantageous combination of traits that align with the specific constraints of closed-loop bioregenerative systems, including limited space, low resource availability, and the need for continuous nutrient cycling. The increasing interest and progress in insect rearing, waste transformation, and nutrient recovery within Earth-based circular systems provide important conceptual and technical insights for the development of space-based systems (Berggren et al., 2025).

### 4.3 Nutritional potential of edible insects

Among the strongest arguments for incorporating insects into BLSS is their high nutritional value, particularly in terms of protein quality, essential fatty acids, and micronutrient content. As summarised in Supplementary Table S2, EU-approved edible species such as *A*. *domesticus* (house cricket), *T*. *molitor* (yellow mealworm), *Alphitobius diaperinus* (lesser mealworm), and *Locusta migratoria* (migratory locust) exhibit nutrient profiles compatible with long-term human dietary needs. On a dry matter basis, these species contain 53%–70% crude protein with high digestibility and favourable amino acid profiles, including lysine, methionine, and tryptophan - amino acids often limited in plant-based diets. Lipid content varies by species, with *T. molitor* and *A. diaperinus* being notably rich in fat (30%–34% DM), including essential fatty acids such as linoleic and α-linolenic acid. Micronutrient concentrations are also promising, with notable levels of iron, zinc, calcium, and vitamin B12. many species are well-liked for their taste (Gómez-Corona and Valentin, 2023). These features make insects particularly well-suited as dietary components for space missions.

### 4.4 Rearing efficiency and space suitability

In addition to their nutritional advantages, insects are highly attractive for integration into Bioregenerative Life Support Systems (BLSS) due to their minimal spatial requirements, and flexible production setups (Berggren et al., 2019). They can be reared in modular vertical systems that are space-efficient and amenable to automation (van Huis et al., 2013). Several edible insect species also exhibit life history traits well-suited to the logistical demands of BLSS. *A. domesticus* matures within 6–8 weeks under optimal conditions, with females capable of laying 1,200–1,500 eggs over their lifespan, supporting scalable and continuous production. *T. molitor* has a somewhat longer life cycle, typically 10–12 weeks from egg to adult, with females producing around 300–500 eggs. Its prolonged larval stage (8–10 weeks) is advantageous for accumulating harvestable biomass. *A. diaperinus* has a generation time of approximately 6–8 weeks and produces 200–400 eggs per female, with rearing conditions similar to *T. molitor* but a slightly faster development rate. *L. migratoria* although requiring more space and social housing due to its gregarious behaviour, completes its life cycle in approximately 2.5–3 months. Females lay multiple egg pods containing 50–80 eggs each, making the species suitable for structured batch production. Together, these traits support flexible rearing strategies including both continuous and staggered harvest cycles making these insect species highly compatible with high-efficiency, resource-constrained systems such as those envisioned for BLSS.

### 4.5 Recycling of nutrients

Critically, insects can convert low-value biomass including inedible plant material, crop residues, and food scraps into high-quality protein. This capacity to utilise organic waste streams offers a direct, biologically mediated alternative to microbial digestion processes and helps close nutrient loops (Miech e al. 2016; Torok et al., 2021). In doing so, insects function as living bioconversion agents, reducing system complexity while fulfilling essential waste transformation roles (Cammack et al., 2021). Lastly, they are extremely efficient in converting feed to body mass (van Huis et al., 2013). This ability is particularly relevant in space missions, where maximising resource recovery and minimising inputs are operational imperatives.

### 4.6 Secondary ecosystem services

Beyond food production and waste conversion, insects provide secondary ecological services. Their frass, a mix of faeces and exuviae, is increasingly recognised as a valuable biofertiliser that can support plant growth and microbial activity. Studies have demonstrated that frass contains bioavailable nutrients and may enhance plant resilience to pathogens (Cammack et al., 2021; Poveda, 2021; Capitán et al., 2025). This contributes to the reintegration of nutrients within the system and enhances plant system functionality.

### 4.7 System integration and adaptation

Functionally, insects are well-suited to the simplified yet interdependent ecological architectures of BLSS. Their multifunctionality combining roles as decomposers, protein producers, and fertiliser providers means they can have many roles in these systems. This could help to maintain ecosystem resilience in minimalist configurations (Berggren et al., 2025). Their growth, reproduction, and nutrient composition can also be modulated by environmental factors such as temperature, humidity, and substrate composition (van Huis, 2021), providing operational flexibility for system management. Even though studies on insects as part of circular food systems are still extremely limited, there has been a large increase in studies during the last 10 years on different aspects of insect rearing and insect ecology important for food production (Boukid et al., 2022), also within the context of sustainability (Berggren et al., 2019). The knowledge already gathered, on different insect species’ growth and survival on different types of feed and suitable housing densities during rearing, will be an extremely valuable resource for developing sustainable BLSS.

## 5 Current knowledge gaps for food production in BLSS

### 5.1 Ecological complexity and functional gaps in simplified systems

As highlighted throughout this review, there are fundamental gaps in knowledge that hinder the construction of BLSS capable of long-term stability (Liu et al., 2021). Although BLSS are intentional simplified compared to terrestrial ecosystems, potentially making them easier to design and maintain, this very simplification introduces unique ecological and functional challenges. One critical challenge lies in the incomplete understanding of how many species function and interact in their natural environments. Even for well-studied biological processes on Earth, significant knowledge gaps persist (Kennedy et al., 2020). A central issue is that the expression of functional traits across different species is often poorly understood (Wohlgemuth et al., 2016), meaning that we often do not know which organisms are responsible for which ecosystem functions. As a result, when specific ecosystem services are replicated in a BLSS context, essential processes may be inadvertently absent or not enough supported. This is especially problematic if the organisms performing key functions in natural systems are either missing or underrepresented in the BLSS. It means that functional redundancy is difficult to achieve in the systems and a loss of failure of a single species could critically destabilise the systems.

### 5.2 Insect physiology and ecology gaps

**FIGURE 3:**
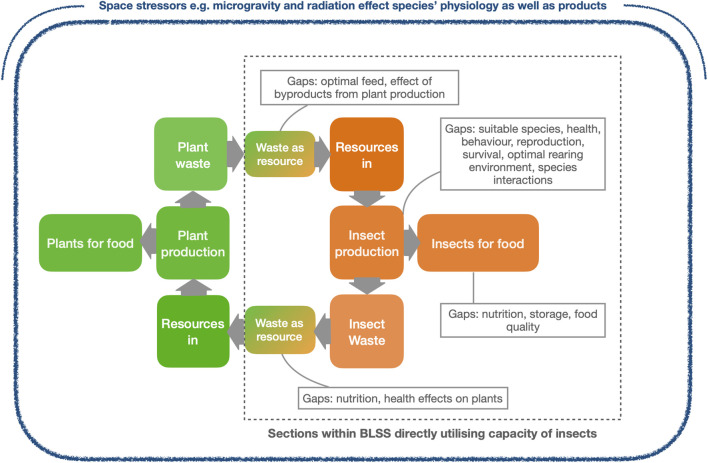
Conceptual model of insect integration in Bioregenerative Life Support Systems (BLSS). Insects serve as multifunctional system components by converting organic waste into edible biomass, producing frass usable as fertiliser for plants, and interacting with microbial communities. These roles contribute to nutrient cycling, ecological resilience, and food production within closed-loop systems. Main gaps in ecological understanding for the insect sections are highlighted. Space environmental stressors affect the system but are not part of the current study.

Insects present a promising solution to the challenge of sustainable nutrition during long-duration spaceflight due to their small size, high feed conversion efficiency, short life cycles, and capacity for bioconversion in bioregenerative life support systems (BLSS). However, despite their potential, a number of key ecological and physiological knowledge gaps limit their successful integration into these systems. To date, the majority of studies focusing on insects within BLSS have lacked experimental data on most aspects of species ecology such as requirements for long-term survival, reproduction, and other important biological factors that influence insect health and behaviour (see Figure 3 for knowledge gaps). The limited knowledge that does exist has largely been generated under terrestrial conditions and needs to be verified under actual or simulated space conditions, a step that very few studies have taken so far.

In addition to this general lack of ecological studies in BLSS, there are several specific and pressing knowledge gaps that must be addressed if insects are to be implemented as multifunctional components in future systems: First, while the nutritional potential of many edible insect species is increasingly well documented, we still lack fundamental knowledge about how these species perform under space-relevant abiotic conditions such as microgravity, radiation exposure and atmospheric variation (Berggren et al., 2025). Microgravity in particular represents a major environmental shift from terrestrial conditions, with profound potential impacts on biological systems. It can influence development, physiology, and behaviour across taxa. Although direct studies on insects are limited, space-based research on model invertebrates such as *Drosophila melanogaster*, *Caenorhabditis elegans*, and tardigrades has shown that exposure to microgravity can alter immune function, circadian rhythms, metabolism, and reproduction (Iyer et al., 2022; Gilbert, 2020). Such alterations in insect metabolism could, in turn, lead to changes in nutrient composition and the stability of insect-derived foods - posing challenges for their use as a consistent nutritional resource.

Second, a deeper understanding of species-specific requirements under closed-system conditions is essential. These include optimal feed sources, suitable humidity and temperature thresholds, and the identification of appropriate rearing densities for long-term sustainability in space habitats. Third, the role of insect–microbe interactions which are known to influence digestion, immunity and waste conversion is largely unexplored in BLSS-relevant contexts. These interactions could significantly affect the reliability and resilience of insect performance within integrated systems, especially in closed and resource-limited environments.

Without addressing these ecological and physiological unknowns, attempts to scale up insect production in space-based systems may encounter unforeseen and potentially critical failures. Therefore, targeted experimental work ideally under simulated or real space conditions is urgently needed to fill these gaps. Only with this knowledge can insects be fully and confidently integrated into BLSS as multifunctional contributors to food production, waste conversion, and nutrient cycling.

## 6 Path forward for insect selection in BLSS

A major limitation in identifying optimal insect candidates for Bioregenerative Life Support Systems is the absence of data on how these organisms respond to the unique abiotic stresses encountered in space, such as microgravity, radiation, and highly controlled atmospheric conditions (Berggren et al., 2023; 2025; Berggren et al., 2025). Although numerous insect species have demonstrated functional value within Earth-based circular food systems (van Huis et al., 2013), translating these findings into space environments remains speculative without targeted, context-specific experimentation. Recognising this limitation is essential to set realistic expectations for future system design. To move the field forward, we propose a conceptual framework that can guide future evaluations of insect suitability for BLSS. This framework emphasises a multidimensional approach, considering each species’ (1) nutritional composition, (2) physiological resilience to environmental stressors, and their function in a system as (3) converters of organic waste and (4) producers of nutrients for other species, such as plants.

To advance the integration of insects into BLSS, targeted experimental frameworks should be established that simulate space-relevant conditions such as microgravity, elevated radiation levels, and controlled atmospheric composition. Based on currently available data from terrestrial research, several insect species emerge as promising candidates for integration into BLSS. *T*. *molitor* (yellow mealworm) is notable for its high protein content, efficient feed-to-biomass conversion, and valuable frass byproduct. *A*. *domesticus* (house cricket) offers a nutrient-dense profile and ease of rearing across a variety of substrates, where frass also have shown to positively affect plants. *Bombyx mori* (silkworm), although more specialised in its dietary needs, has historical precedence in BLSS experiments and may provide insight into insect-microbe-plant dynamics. While these species span different ecological niches and functional traits, they collectively represent a spectrum of services very interesting to closed-loop life support. Given the limited empirical evidence from space-specific contexts, we suggest a phased research agenda that begins with terrestrial experiments of candidate species that should test insect performance under simulated space conditions, such as microgravity analogues and radiation exposure. These studies should aim to assess not only individual survival, growth, and reproduction, but also how insects interact with other species such as plants and microbial communities. Experimental modules could include small-scale, controlled habitats with integrated waste and plant systems to test inter-species dynamics and nutrient flow. Incorporating real-time monitoring of behaviour, microbiome shifts, and physiological stress responses will provide valuable insights into adaptation and resilience. An increased ecological understanding of focus species would increase the ability to build resilience in the BLSS. Through such approach, we can develop an evidence-based, adaptable methodology for selecting insect species that are not only nutritionally and ecologically valuable but also physiologically suited to the constraints of space habitation.

## 7 Conclusion

Currently, true circular system approaches are still largely lacking in terms of experiments, and this is the case for both space and Earth applications. Even when a complete circular approach is modelled, relevant biological questions are very rarely included. The lack of studies focusing on ecological aspects of interacting organisms and their subsequent functions likely results from current and previous research prioritisations. However, stable and functioning food production in BLSS is a critical foundation for long space travels and planetary colonisations. Therefore, there is a need to increase the amount of research that focuses on how nutritious food can be produced, with this requiring the incorporation of sound ecological knowledge to understand the biological requirements for the organisms that will provide these services. Based on this review of the current literature, we suggest that for BLSS to provide long-term food production, research priorities need to include: (1) examining promising animal taxa, such as insects, that can provide multiple services within these systems (e.g., food production and waste recycling), (2) basic biological attributes of these species, including relevant physiology, reproduction, behaviour, health, and nutrient content, (3) use current development of Earth-based circular food systems to inform which species and processes should be prioritised (here insects appear to be good candidates for BLSS), and (4) investigate whether the biological processes and functions these animals provide in terrestrial systems, translates into real space conditions by performing trials in actual or simulated space conditions. Given their multifunctional roles, adaptability, and compatibility with circular systems, insects are a uniquely valuable candidate for integrated food and waste management in BLSS, warranting priority in future research agendas.
