# Supplementary material for: Insects in bioregenerative life support systems: unlocking their role in space sustainability
Source: Front Physiol. 2025 Sep 10;16:1621099. doi: 10.3389/fphys.2025.1621099 (PMC12457449; doi:10.3389/fphys.2025.1621099)
Supplement: Supplementary file 1 [file Table1.pdf]

Table S1. The pubished 280 scientific papers on Bioregenerative Life Support Systems used for analysis in the study. From these papers data were extracted on study type, primary focus, and whether plants or animals were used, including the species involved. I was also recorded whether studies examined species interactions and the species were involved.

Authors	Title	Publication Year	Journal	Volume	Pages
M. Masukawa, T. Ochiai, S. Kamigaichi, N. Ishioka, S. Uchida, Y. Kono, T. Sakimura	Investigation of Photosynthetic CO_2 Fixation Efficiency of the Microalga Chlorella sp. under Microgravity on the ISS	2018	Life Sciences in Space Research	17	64-71
Ariel M. Hughes, Joshua P. Vandenbrink, John Z. Kiss	Gravitropism and the role of the actin cytoskeleton in plants exposed to spaceflight environments	2021	Life	11	320
Y. Kubo, F. Takasu, R. Shimura, S. Nagaoka, N. Shigesada	Model analysis of an artificial ecosystem for material circulation control	2001	Ecological Modelling	142	269-286
A.C. Schuerger, C.S. Brown	Spectral quality affects phytochrome-controlled growth and productivity of radish in controlled environments	1997	HortScience	32	129-132

M. Niihori; Y. Mogami; K. Naruse; S.A. Baba	Effects of gravity and light on the chloroplast arrangement in Lemna gibba	2004	Biological Sciences in Space	18	208-209
Williamson, M.	Lunar exploration and development—A sustainable model	2005	Acta Astronautica	57	161–166
A.A. Ermash	Photosynthetic systems as components of a bioregenerative life support system	2007	Acta Astronautica	60	608-614
Miguel A. Valbuena; Aranzazu Manzano; Joshua P. Vandenbrink; Veronica Pereda-Loth; Eugenie Carnero-Diaz; Richard E. Edelmann; John Z. Kiss	Arabidopsis thaliana root phototropism and gravitropism are differently affected by red light and microgravity	2020	Plants	9	949
R. Repaske, R. Mayer	Dense Autotrophic Cultures of Alcaligenes eutrophus	1976	Applied and Environmental Microbiology	32	592-597
M.S. Ruff; D.T. Krizek; R.M. Mirecki; D.W. Inouye	Comparison of hydroponic and soil systems for lettuce growth: Effects on photosynthesis and biomass production	1987	Journal of the American Society for Horticultural Science	112	494-498

R.M. Wheeler; C.L. Mackowiak; J.C. Sager; W.M. Knott; C.R. Hinkle	Potato growth and yield using nutrient film technique (NFT)	1990	American Potato Journal	67	177-187
D.G. Mortley; P.A. Loretan; W.A. Hill; C.K. Bonsi; C.E. Morris; R. Hall; D. Sullen	Biomass accumulation and yield of sweetpotato using nutrient film technique	1991	HortScience	26	261-263
C.L. Mackowiak; R.M. Wheeler	Effect of CO2 concentration on gas exchange rates and biomass accumulation of wheat grown in a closed plant growth chamber	1991	HortScience	26	1204-1207
Straight, C.L.; Bubenheim, D.L.; Bates, M.E.; Flynn, M.T.	The CELSS Antarctic Analog Project: A Validation of CELSS Methodologies at the South Pole Station	1993	SAE Transactions	102	1448-1459
D.G. Mortley; C.K. Bonsi; P.A. Loretan; W.A. Hill; C.E. Morris	Comparative yield of sweetpotato using different hydroponic systems	1994	HortScience	29	258-259
T.W. Tibbitts; W. Cao; S.M. Bennett	Growth of potatoes for CELSS applications: Productivity under different photoperiods and radiation levels	1994	Advances in Space Research	14	345-350

Bubenheim, D.; Wignarajah, K.; Berry, W.; Wydeven, T.	Phytotoxic Effects of Gray Water Due to Surfactants	1997	Journal of the American Society for Horticultural Science	122	792-796
Goto, E.; Iwabuchi, K.; Takakura, T.	Effect of Reduced Total Air Pressure on Spinach Growth	1995	Journal of Agricultural Meteorology	51	139–143
J.H. Batten; G.W. Stutte; R.M. Wheeler	Use of porous tube nutrient delivery systems for vegetable crops in a CELSS	1995	Advances in Space Research	16	85-88
Wheeler, RM; Mackowiak, CL; Stutte, GW; Sager, JC; Yorio, NC; Ruffe, LM; Fortson, RE; Dreschel, TW; Knott, WM; Corey, KA	NASA'S Biomass Production Chamber: A Testbed for Bioregenerative Life Support Studies	1996	Advances in Space Research	18	215-224
Wheeler, RM; Mackowiak, CL; Sager, JC; Yorio, NC; Knott, WM; Berry, WL	Growth and gas exchange by wheat in a large, closed chamber	1996	Advances in Space Research	18	259-268
Mackowiak, CL; Wheeler, RM; Stutte, GW; Yorio, NC; Sager, JC	Use of a salad machine for life support system plant production testing	1996	Advances in Space Research	18	281-288

C.F. Johnson; T.W. Dreschel; C.S. Brown; R.M. Wheeler	Plant growth on a porous tube nutrient delivery system	1996	Life Support & Biosphere Science	3	53-59
C.S. Brown; W.M. Cox; T.W. Dreschel; P.V. Chetirkin	Air distribution and carbon dioxide removal in a spacecraft plant growth chamber	1996	Life Support & Biosphere Science	3	71-76
Wu, W.H.; Lu, J.Y.; Jones, A.R.; Mortley, D.G.; Loretan, P.A.; Bonsi, C.K.; Hill, W.A.	Proximate Composition, Amino Acid Profile, Fatty Acid Composition, and Mineral Content of Peanut Seeds Hydroponically Grown at Elevated CO2 Levels	1997	J. Agric. Food Chem.	45	3863−3866
Reuveni, J.; Bugbee, B.	Very High CO2 Reduces Photosynthesis, Dark Respiration and Yield in Wheat	1997	Annals of Botany	80	539–546
Goins, G.D.; Yorio, N.C.; Sanwo, M.M.; Brown, C.S.	Photomorphogenesis, photosynthesis, and seed yield of wheat plants grown under red light-emitting diodes (LEDs) with and without supplemental blue lighting	1997	Journal of Experimental Botany	48	1407-1413
Kliss, M; Heyenga, AG; Hoehn, A; Stodieck, LS	Recent advances in technologies required for a “Salad Machine”	2000	Advances in Space Research	26	263-269

Savage, CJ; Tan, GBT; Lasseur, C	ESA Developments in Life Support Technology: Achievements and Future Priorities	2001	Acta Astronautica	49	331-344
A. Tani; T. Okuma; E. Goto; Y. Kitaya; T. Saito; H. Takahashi	Ground performance of air conditioning and water recycle system for a space plant box	2001	Advances in Space Research	27	1557-1562
P. Kostov; T. Ivanova; I. Dandolov; S. Sapunova; I. Ilieva	Adaptive Environmental Control for Optimal Results during Plant Microgravity Experiments	2002	Acta Astronautica	51	213-220
D. L. Bubenheim, G. Schlick, D. Wilson, M. Bates	Performance of the CELSS Antarctic Analog Project (CAAP) Crop Production System	2003	Advances in Space Research	31	255-262
Kitaya, Y; Shibuya, T; Yoshida, M; Kiyota, M	Growth and gas exchange of plants in a closed air-conditioned greenhouse for space agriculture	2004	Advances in Space Research	34	1466-1469
Shen Yunze, Guo Shuangsheng	Numerical simulation of biological waste water treatment with aerated submerged filters	2004	Journal of Environmental Sciences	16	677-682

Nelson, M; Dempster, WF; Silverstone, S; Alling, A; Allen, JP; van Thillo, M	Crop yields and light/energy efficiency in a closed ecological system: Laboratory Biosphere experiments with wheat and sweet potato	2005	Advances in Space Research	35	1539-1543
Gary W. Stutte, N.C. Yorio, S.L. Edney, J.T. Richards, M.P. Hummerick, M. Stasiak, M. Dixon, R.M. Wheeler	Photosynthetic photon flux, photoperiod, and plant growth: Implications for space-based plant production systems	2005	Advances in Space Research	35	1809-1818
Stutte, G.W.; Monje, O.; Hatfield, R.D.; Paul, A.-L.; Ferl, R.J.; Simone, C.G.	Microgravity effects on leaf morphology, cell structure, carbon metabolism and mRNA expression of dwarf wheat	2006	Planta	224	1038–1049
Gaohong Wang; Yongding Liu; Genbao Li; Chunxiang Hu; Delu Zhang; Xiaoyan Li	Effects of space flight on photosynthetic efficiency and ultrastructure of Anabaena sp. PCC7120	2006	Advances in Space Research	38	1235-1240
Xu Chunxiao; Liu Hong	Crop candidates for the bioregenerative life support systems in China	2008	Acta Astronautica	63	1076-1080
Hui Liu; Minjuan Wang; Yuming Fu; Hong Liu	Effects of Different Light Qualities on Plant Growth, Gas Exchange and Water-Use Efficiency in Wheat (Triticum aestivum L.)	2008	Advances in Space Research	41	1289-1294

De Micco, Veronica; Aronne, Giovanna; Colla, Giuseppe; Fortezza, Raimondo; De Pascale, Stefania	Agro-biology for bioregenerative Life Support Systems in long-term Space missions: General constraints and the Italian efforts	2009	Journal of Plant Interactions	4	241-252
Young, R.C.; Buttner, W.J.; Linnell, B.R.; Ramesham, R.	Hydrogen sensors for safety monitoring in hydrogen and fuel cell applications	2011	International Journal of Hydrogen Energy	36	2462-2472
Yongkang Tang; Yunze Shen; Hongqi Feng; Hao Wu; Ruixin Mao; Weidang Ai; Zhiqiang Wu	Effects of different nitrogen forms on lettuce growth and nitrate accumulation in hydroponics	2013	HortScience	48	1125-1129
Ting Zhao, Guanghui Liu, Dianlei Liu, Yue Yi, Beizhen Xie, Hong Liu	Effects of simulated microgravity on photosynthesis and related gene expression of alfalfa	2014	Acta Astronautica	94	253-259
Ting Wang; Wei Xu; Huasheng Li; Chenguang Deng; Hui Zhao; Yuejin Wu; Min Liu; Lijun Wu; Jinying Lu; Po Bian	Effects of Microgravity on the Photosynthetic Characteristics of Wheat in Spaceflight	2014	Advances in Space Research	53	1574-1580
Angiola Desiderio; Anna Maria Salzano; Andrea Scaloni; Silvia Massa; Maria Pimpinella; Vanessa De Coste; Claudio Pioli; Luca Nardi; Benvenuto	Proteomic Analysis of Daucus carota L. Seedlings Exposed to Simulated Microgravity	2014	Proteomics	14	1001-1011

Shen, Y.Z.; Guo, S.S.; Ai, W.D.; Tang, Y.K.	Effects of illuminants and illumination time on lettuce growth, yield and nutritional quality in a controlled environment	2014	Life Sciences in Space Research	2	38–42
Massa, G.; Graham, T.; Haire, T.; Flemming, C. II; Newsham, G.; Wheeler, R.	Light-emitting Diode Light Transmission through Leaf Tissue of Seven Different Crops	2015	HortScience	50	501–506
Yu.A. Berkovich; N.M. Krivobok; A.S. Krivobok; S.O. Smolyanina	Advanced nutrient root-feeding system for conveyor-type cylindrical plant growth facilities for microgravity	2016	Life Sciences in Space Research	8	14-21
Dong, Chen; Shao, Lingzhi; Fu, Yuming; Wang, X.	Nutrient removal in microalgae-based wastewater treatment systems for space bioregenerative life support	2017	Acta Astronautica	140	91-98
Villani, Maria Elena; Massa, Silvia; Lopresto, Vanni; Pinto, Rosanna; Salzano, Anna Maria; Scaloni, Andrea; Benvenuto, Eugenio; Desiderio, Angiola	Effects of high-intensity static magnetic fields on a root-based bioreactor system for space applications	2017	Life Sciences in Space Research	15	79-87
Li, Ting; Zhang, Liangchang; Ai, Weidang; Dong, Wenyi; Yu, Qingni	A modified MBR system with post advanced purification for domestic water supply system in 180-day CELSS: Construction, pollutant removal and water allocation	2018	Journal of Environmental Management	222	37-43

Ivo Bertalan; Dania Esposito; Giuseppe Torzillo; Cecilia Faraloni; Udo Johanningmeier; Maria Teresa Giardi	Involvement of Photosynthetic Microorganisms in Regenerative Life Support Systems: The Challenge of Space	2018	Life Sciences in Space Research	17	24-30
Xun, W.; Yang, D.; Huang, Z.; Sha, H.; Chang, H.	Cellular immunity monitoring in long-duration spaceflights based on an automatic miniature flow cytometer	2018	Sensors and Actuators B: Chemical	267	419–429
Shen, Y.; Guo, S.; Zhao, P.; Wang, L.; Wang, X.; Li, J.; Bian, Q.	Research on lettuce growth technology onboard Chinese Tiangong II Spacelab	2018	Acta Astronautica	144	97–102
Dong, Chen; Fu, Yuming; Liu, Guanghui; Liu, Hong	Effects of light intensity and nitrogen concentration on nitrogen transformation and microalgae growth in closed photobioreactors	2020	Science of the Total Environment	723	137934
Jie Wang; Jufang Wang; Wenjian Li; Guanghong Luo; Songqi Yang; Yan Du; Wei Wei; Wenjie Jin; Shanwei Luo; Xin Li	Optimization of Water Use Efficiency in Lettuce Using Supplemental Red Light in Spaceflight	2020	Life Sciences in Space Research	25	01-aug
N.V. Zaimenko; B.O. Ivanytska; N.V. Rositska; N.P. Didyk; D. Liu; M. Pyzyk; J. Slaski	Structural and Functional Peculiarities of Plants under Microgravity and Spaceflight Conditions	2020	Life Sciences in Space Research	27	30-40

Romano, Leone Ermes; Aronne, Giovanna	The World Smallest Plants (Wolffia Sp.) as Potential Species for Bioregenerative Life Support Systems in Space	2021	Plants	10	1896
Christina M. Johnson, Haley O. Boles, LaShelle E. Spencer, Lucie Poulet, Matthew Romeyn, Jess M. Bunchek, Ralph Fritsche, Gioia D. Massa, Aubrie O'Rourke, Raymond M. Wheeler	Supplemental Food Production With Plants: A Review of NASA Research	2021	Frontiers in Astronomy and Space Sciences	8	734343
P.R. Richter, Y. Liu, Y. An, X. Li, A. Nasir, S.M. Strauch, I. Becker, J. Kurger, M. Schuster, M. Ntefidou, V. Daiker, F.W.M. Haag, A. Aiach, M. Lebert	Spaceflight activates cell division and cytoskeleton reorganization in Euglena gracilis	2022	International Journal of Molecular Sciences	23	8320
Chenjunnan Zhou; Zhao Xu; Lingxiao Wang; Rulu Qiao; Yanming Li; Ruixue Chang; Weidang Ai	Lettuce growth and photosynthetic characteristics under different light intensities and spectral qualities in a hydroponic system	2022	Plants	11	2954
Antonio Pannico, Gionata Cimini, Claudia Quadri, Roberta Paradiso, Lorenzo Bucchieri, Youssef Rouphael, Stefania De Pascale	Plant growth and quality of two lettuce cultivars grown in a closed cultivation system: The role of photosynthetic photon flux density and spectral quality	2023	Frontiers in Plant Science	14	1187698
Caporale, A.G.; Paradiso, R.; Liuzzi, G.; Palladino, M.; Amitrano, C.; Arena, C.; Arouna, N.; Verrillo, M.; Cozzolino, V.; De Pascale, S.; Adamo, P.	Green compost amendment improves potato plant performance on Mars regolith simulant as substrate for cultivation in space	2023	Plant and Soil	486	217–233

Tong, F.; Wu, C.; Wang, L.; Jing, X.; Wu, S.; Sun, J.; Hu, Y.; Li, J.; Wang, Y.; Li, Y.	Can XunTian Tai Chi intervention improve the level of emotional regulation of crew members in the Controlled Ecological Life Support System?	2023	Sports Medicine and Health Science	5	245–250
Su, S.-H.; Levine, H.G.; Masson, P.H.	Brachypodium distachyon Seedlings Display Accession-Specific Morphological and Transcriptomic Responses to the Microgravity Environment of the International Space Station	2023	Life	13	626
Liistro, Elisabetta; Battistuzzi, Mariano; Cocola, Lorenzo; Claudi, Riccardo; Poletto, Luca; La Rocca, Nicoletta	Synechococcus sp. PCC7335 responses to far-red enriched spectra and anoxic/microoxic atmospheres: Potential for astrobiotechnological applications	2024	Plant Physiology and Biochemistry	213	108793
Romano, L.E.; van Loon, J.J.W.A.; Vincent-Bonnieu, S.; Aronne, G.	Wolffia globosa, a novel crop species for protein production in space agriculture	2024	Scientific Reports	14	27979
MacElroy, R D; Tremor, J; Smernoff, D T; Knott, W; Prince, R P	A review of recent activities in the NASA CELSS program	1987	Advances in Space Research	7	53-57
Trotman, AA; Almazan, AM; Alexander, AD; Loretan, PA; Zhou, X; Lu, JY	Biological Degradation and Composition of Inedible Sweetpotato Biomass	1996	Advances in Space Research	18	257-279

Ivanova, TN; Kostov, PT; Sapunova, SM; Dandolov, IW; Salisbury, FB; Bingham, GE; Sytchov, VN; Levinskikh, MA; Podolski, IG; Bubenheim, DB; Jahns, G.	Six-month space greenhouse experiments - A step to creation of future biological life support systems	1998	Acta Astronautica	42	nov-23
M.A. Benjaminson; S. Lehrer; D.A. Macklin	Bioconversion Systems for Food and Water on Long Term Space Missions	1998	Acta Astronautica	43	329-348
V.N. Sychev; E.Ya. Shepelev; G.I. Meleshko; T.S. Gurieva; M.A. Levinskikh; I.G. Podolsky; O.A. Dadasheva; V.V. Popov	Main Characteristics of Biological Components of Developing Life Support System Observed During the Experiments Aboard Orbital Complex MIR	2001	Advances in Space Research	27	1529-1534
Tirranen, L.S.; Borodina, E.V.; Ushakova, S.A.; Rygalov, V.Y.; Gitelson, J.I.	Effect of volatile metabolites of dill, radish and garlic on growth of bacteria	2001	Acta Astronautica	49	105–108
Slenzka, K.	Life Support for Aquatic Species - Past; Present; Future	2002	Advances in Space Research	30	789-795
Berkovich, Y.A.; Krivobok, N.M.; Sinyak, Y.Y.; Smolyanina, S.O.; Grigoriev, Y.I.; Romanov, S.Y.; Guissenberg, A.S.	Developing a vitamin greenhouse for the life support system of the international space station and for future interplanetary missions	2004	Advances in Space Research	34	1552–1557

Silverstone, S; Nelson, M; Alling, A; Allen, JP	Soil and crop management experiments in the Laboratory Biosphere: An analogue system for the Mars on Earth facility	2005	Advances in Space Research	35	1544–1551
Yu, XiaoHui; Liu, Hong; Tong, Ling	Feeding scenario of the silkworm Bombyx Mori, L. in the BLSS	2008	Acta Astronautica	63	1086–1092
J.L. Garland; K.L. Cook; M. Johnson; R. Sumner; N. Fields	Microbial Ecology and the Stability of Waste Processing Bioreactors in Space	2010	Microbial Ecology	59	183-195
Yang, Y.; Tang, L.; Tong, L.; Liu, Y.; Liu, H.; Li, X.	Initial ground experiments of silkworm cultures living on different feedstock for provision of high quality animal protein for human in space	2010	Advances in Space Research	46	707–711
L. Tong, X. Yu, H. Liu	Insect food for astronauts: gas exchange in silkworms fed on mulberry and lettuce and the nutritional value of these insects for human consumption during deep space flights	2011	Bulletin of Entomological Research	101	613-622
Liang, Xue; Fu, Yuming; Liu, Hong	Isolation and characterization of enzyme-producing bacteria of the silkworm larval gut in bioregenerative life support system	2015	Acta Astronautica	116	247–253

Li, X.; Richter, P.R.; Hao, Z.; An, Y.; Wang, G.; Li, D.; Liu, Y.; Strauch, S.M.; Schuster, M.; Haag, F.W.; Lebert, M.	Operation of an enclosed aquatic ecosystem in the Shenzhou-8 mission	2017	Acta Astronautica	134	17-22
Jin, Xiangdan; Ai, Weidang; Li, Chengxian; Zhang, Liangchang; Yu, Qingni; Tang, Yongkang; Dong, Wenyi	Operation overview of a biological waste treatment system during the 4-crew 180-day integrated experiment in the controlled ecological life support system (CELSS)	2021	Life Sciences in Space Research	29	15-21
Schuerger, Andrew C.; Amaradasa, Bimal S.; Dufault, Nicholas S.; Hummerick, Mary E.; Richards, Jeffrey T.; Khodadad, Christina L.; Smith, Trent M.; Massa, Gioia D.	Fusarium oxysporum as an Opportunistic Fungal Pathogen on Zinnia hybrida Plants Grown on board the International Space Station	2021	Astrobiology	21	1029-1048
Knie, Miriam; Schoppmann, Kathrin; Eck, Hendrik; Ribeiro, Bernard Wolfschoon; Laforsch, Christian	The potential of edible insects in bioregenerative life support systems	2022	Acta Astronautica	196	649-659
Jin, Xiangdan; Ai, Weidang; Zhang, Yang; Dong, Wenyi	Application of functional microbial agent in aerobic composting of wheat straw for waste recycling	2022	Life Sciences in Space Research	33	13-20
Aronne, G.; Muthert, L.W.F.; Izzo, L.G.; Romano, L.E.; Iovane, M.; Capozzi, F.; Manzano, A.; Ciska, M.; Herranz, R.; Medina, F.J.; Kiss, J.Z.; van Loon, J.J.W.A.	A novel device to study altered gravity and light interactions in seedling tropisms	2022	Life Sciences in Space Research	32	8–16

Kitaya, Y.; Kawamoto, T.; Endo, R.; Shibuya, T.	Effect of fish density on biological production in aquaponics combining lettuce hydroponics and loach aquaculture for controlled ecological life support systems in space	2023	Frontiers in Astronomy and Space Sciences	10	1197402
Romano, Donato; Di Giovanni, Adriano; Stefanini, Cesare	Assessing black soldier fly pupation and survival in lunar regolith simulant	2024	Acta Astronautica	223	505–511
Zaidi, M.A.; Murase, H.; Nishiura, Y.; Takigawa, H.; Honami, N.; Tani, A.	Development of centrifugal phytotron to study the gravity effect on vegetable plant growth	1996	Acta Horticulturae	440	70-74
Iwabuchi, K.; Goto, E.; Takakura, T.	Effect of vapor pressure deficit on spinach growth under hypobaric conditions	1996	Acta Horticulturae	440	60-63
Spurlock, J.M.; Modell, M.	Technology Requirements for Nonterrestrial Ecosystems	1978	NASA Conference Publication	17	27-35
Skoog, A I	Progress in European CELSS Activities	1987	NASA Conference Publication	7	06-mars

Petersen, GR; Seshan, PK; Dunlop, EH	Algae air revitalization for CELSS: A feasibility study of culture growth in microgravity	1987	Advances in Space Research	7	125-132
Belkin, S.; Mehlhorn, R.J.; Packer, L.	Proton Gradients in Intact Cyanobacteria	1987	Plant Physiology	84	25-30
GUPTA, G	Water Purification in a Bioregenerative Life Support System Using Aquatic Plants	1988	Advances in Space Research	8(4)	161-164
Salisbury, F B; Bugbee, B; Bubenheim, D	Wheat production in controlled environments for use in a CELSS	1988	NASA Contractor Report	7	123-132
Volk, T; Rummel, J D	Transpiration During Life Cycle in Controlled Wheat Growth	1989	Advances in Space Research	9(8)	61-64
Mori, K; Ohya, H; Matsumoto, K; Furune, H	Sunlight Supply and Gas Exchange in Microalgal Bioreactor	1990	Biological Sciences in Space	4(2)	88-92

Westgate, P; Kohlmann, K; Hendrickson, R; Ladisch, M R	Bioprocessing in Space	1992	Enzyme and Microbial Technology	14	76-79
Oguchi, M.; Otsubo, K.; Nitta, K.; Hatayama, S.	Development of a flight experiment system for Closed Ecological Life Support System in space	1992	Advances in Space Research	12	65-74
Yandell, B.S.; Najar, A.; Wheeler, R.; Tibbitts, T.W.	Comparison of potato growth models with experimental data for plants grown at elevated CO_2	1992	Acta Horticulturae	313	239-246
Y.A. Berkovich; P.V. Chetirkin; R.M. Wheeler; J.C. Sager	Advanced oxygen production technologies for closed ecological life support systems (CELSS)	1994	Advances in Space Research	14	403-411
Fu, B.; Nelson, P.E.; Irvine, R.; Kanach, L.L.	Processing of Nutritious, Safe and Acceptable Foods from CELSS Candidate Crops	1996	Advances in Space Research	18	241-248
Salisbury, F.B.; Clark, M.A.Z.	Suggestions for crops for CELSS	1996	Advances in Space Research	18	149-157

Schneegurt, M.A.; Arieli, B.; Nielsen, S.S.; Trumbo, P.R.; Sherman, L.A.	Cyanobacterial Growth on Simulated Wastewater Media: Applications for Space Life Support Systems	1996	Life Support & Biosphere Science	3	45-50
Wheeler, EF; Kossowski, J; Goto, E; Langhans, RW; White, G; Albright, LD; Wilcox, D	Consideration in selecting crops for the human-rated life support system: A linear programming model	1996	Advances in Space Research	18	233-236
R.J. Bula, R.C. Morrow, T.W. Tibbitts	Potato Growth in a Porous Tube Water and Nutrient Delivery System	1996	Advances in Space Research	18	243-249
G.E. Bingham, S.B. Jones, D. Or, I.G. Podolski, M.A. Levinskikh, V.N. Sytchov, T. Ivanova, P. Kostov, S. Sapunova, I. Dandolov, D.B. Bubenheim, G. Jahns	Microgravity effects on water supply and substrate structure in porous ceramic media	1996	Advances in Space Research	18	127-136
G.E. Bingham, F.B. Salisbury, W.F. Campbell, J.G. Carman, D.L. Bubenheim, B. Yendler, V.N. Sytchev, Y.A. Berkovitch, M.A. Levinskikh, I.G. Podolsky	Effect of gravity on plant growth and morphology during the 122-day MIR space station experiment	1996	Advances in Space Research	18	147-156
Berkovitch, Y.A.	INSTRUMENTATION FOR PLANT HEALTH AND GROWTH IN SPACE	1996	Advances in Space Research	18	157-162

Stutte, G W; Mackowiak, C L; Yorio, N C; Wheeler, R M	Theoretical and practical considerations for staggered production of crops in a BLSS	1997	Advances in Space Research	20	1851-1854
Strayer, R.F.; Atkinson, C.F.	Recycling Nutrients from Crop Residues For Space Applications	1997	Compost Science & Utilization	5	25-31
Chun, C; Mitchell, C A	Dynamic optimization of CELSS crop photosynthetic rate by computer-assisted feedback control	1997	Advances in Space Research	20	1855-1860
Chun, C; Mitchell, C A	Crop modeling and optimization of resource use in CELSS: A review	1997	Acta Astronautica	20	1855-1860
D.L. Bubenheim, C. Lewis	Application of NASA's Advanced Life Support Technologies in Polar Regions	1997	Advances in Space Research	20	2037-2044
Yu.A. Berkovich, N.M. Krivobok, Yu.E. Sinyak	Project of conveyer-type space greenhouse for cosmonauts' supply with vitamin greenery	1998	Advances in Space Research	22	1401–1405

T. Grotenhuis, J. Reuveni, B. Bugbee	Super-elevated CO2 enhances photosynthesis and productivity of hydroponic lettuce in continuous light	1998	HortScience	33	1004-1008
Andre, M; Chagvardieff, P	Synergies Between Plant Research Conducted for Terrestrial and for Space Purposes	1999	Advances in Space Research	24	265-269
D.T. Smernoff, R.L. Mancinelli	Use of Higher Plants to Remove CO2 from Controlled Environments	1999	Life Support & Biosphere Science	6	255-262
Drysdale, AE; Rutkze, CJ; Albright, LD; LaDue, RL	Light and CO2 control strategies for bioregenerative life support systems crop production	1999	Life Support & Biosphere Science	6	87–94
C. Kecskes	Solid waste processing and resource recovery for bioregenerative life support systems	1999	Acta Astronautica	44	629-633
Samsonov, NM; Bobe, LS; Gavrilov, LI; Novikov, VM; Farafonov, NS; Grigoriev, JI; Zaitsev, EN; Romanov, SJ; Grogoriev, AI; Sinjak, JE	Long-Duration Space Mission Regenerative Life Support	2000	Acta Astronautica	47	129–138

Jones, SB; Or, D	Microgravity and wetting control in plant root zones	2000	Life Support & Biosphere Science	7	251–261
S.I. Bartsev, V.V. Mezhevikin, V.A. Okhonin	Evaluation of Optimal Configuration of Hybrid Life Support System for Space	2000	Advances in Space Research	26	323-326
K. Slenzka, M. Duenne, B. Jastorff, M. Schirmer	Toxicity testing in aquatic systems for bioregenerative life support system – Using fish as bioindicators	2000	Acta Astronautica	46	665-672
Tani, A.; Seino, K.	Effects of Side Cooling on Temperature, Humidity and Water Recycling Efficiency in a Culture Vessel for a Space Experiment	2000	Environmental Control in Biology	38	79-87
Lötsch, B.	Modeling photosynthetic efficiency and oxygen production for controlled closed bioregenerative life support systems	2001	Life Support and Biosphere Science	8	41–52
Willigert Raatschen, Helmut Preiss	Potential and Benefits of Closed Loop ECLS Systems on the ISS	2001	Acta Astronautica	48	411-419

Y. Sinyak, A. Grigoriev, V. Gaydadimov, T. Gurieva, A. Levinskih, B. Pokrovskii	Spaceflight experiments with higher plants in Russia	2001	Advances in Space Research	27	939-944
S. Lee; R.M. Lueptow	Hydroponic plant growth system model for microgravity environments	2001	Journal of Biomechanical Engineering	123	144-151
Cockell, C.S.	The Martian and Extraterrestrial UV Radiation Environment Part II: Further Considerations on Materials and Design Criteria for Artificial Ecosystems	2001	Acta Astronautica	49	631-640
Jack, D.A.; Nakamura, T.; Sadler, P.; Cuello, J.L.	Evaluation of Two Fiber Optic–Based Solar Collection and Distribution Systems for Advanced Space Life Support	2002	Transactions of the ASAE	45	1547-1558
GH Wang, GB Li, CX Hu, YD Liu, LR Song, GH Tong, XM Liu, ET Cheng	Microalgae as a component of BLSS for oxygen regeneration and food production	2002	Acta Astronautica	51	895-899
B. Yendler	Bioregenerative life support systems: Oxygen production in space	2002	Acta Astronautica	51	745-754

Benjaminson, M.A.; Gilchriest, J.A.; Lorenz, M.	IN VITRO EDIBLE MUSCLE PROTEIN PRODUCTION SYSTEM (MPPS): STAGE 1, FISH	2002	Acta Astronautica	51	879–889
Schlager, KJ	Development of next-generation bioregenerative life support technologies	2003	Advances in Space Research	31	169-176
Hyeon-Hye Kim, Raymond M Wheeler, John C Sager, Neil C Yorio, Gregory D Goins	A computer simulation of lettuce production in controlled ecological life support systems (CELSS) under different scenarios of crop productivity and area allocation	2003	Advances in Space Research	31	179–188
D.H. Fleisher, J. Cavazzoni, G.A. Giacomelli, K.C. Ting	Adaptation of SUBSTOR for controlled-environment potato production with elevated carbon dioxide	2003	Transactions of the ASAE	46	531–538
Y. Kitaya, A. Tani, E. Goto, T. Saito, H. Takahashi	Effects of gravity and CO2 on plant growth and photosynthesis	2003	Advances in Space Research	31	2135-2140
Sutter, B.; Ming, D.W.; Clearfield, A.; Hossner, L.R.	Mineralogical and Chemical Characterization of Iron-, Manganese-, and Copper-Containing Synthetic Hydroxyapatites	2003	Soil Sci. Soc. Am. J.	67	1935–1942

Xu, X.H.; Shi, Y.; Kwak, D.; Chang, S.G.; Fisher, J.W.; Pisharody, S.; Moran, M.J.; Wignarajah, K.	The Use of Rice Hulls for Sustainable Control of NOx Emissions in Deep Space Missions	2003	Ind. Eng. Chem. Res.	42	1813-1820
Xu, X.H.; Shi, Y.; Liu, S.H.; Wang, H.P.; Chang, S.G.; Fisher, J.W.; Pisharody, S.; Moran, M.; Wignarajah, K.	Method for the Control of NOx Emissions in Long-Range Space Travel	2003	Energy & Fuels	17	1303-1310
Rygalov, Vadim Y; Fowler, Philip A; Wheeler, Raymond M; Bucklin, Ray A	Water cycle and its management for plant habitats at reduced pressures	2004	Habitation	10	49-59
Cuello, JL	Design and performance of a new high flux controlled environment agriculture (CEA) system for space applications	2004	Acta Astronautica	55	333-340
Paul, AL; Schuerger, AC; Popp, MP; Richards, JT; Manak, MS; Ferl, RJ	Hypobaric Biology: Arabidopsis Gene Expression at Low Atmospheric Pressure	2004	Plant Physiology	134	215–223
A. Kondyurin, B. Lauke, I. Kondyurina, E. Orba	Creation of biological module for self-regulating ecological system by the way of polymerization of composite materials in free space	2004	Advances in Space Research	34	1585–1591

B.N. Kiforenko, I.Y. Vasil'ev	Physiological and technical fundamentals of plant cultivation in bioregenerative life support systems	2004	Acta Astronautica	54	211-220
G.H. Wang, G.B. Li, D.H. Li, Y.D. Liu, L.R. Song, G.H. Tong, X.M. Liu, E.T. Cheng	Real-time studies on microalgae under microgravity	2004	Acta Astronautica	55	131-137
Sutter, B.; Taylor, R.E.; Hossner, L.R.; Ming, D.W.	Solid State 31Phosphorus Nuclear Magnetic Resonance of Iron-, Manganese-, and Copper-Containing Synthetic Hydroxyapatites	2004	Soil Science Society of America Journal		455-463
T. Ayala-Silva, C.A. Beyl	Vegetable crop production under controlled environments for space missions	2005	HortScience	40	1117-1124
Norikane, J.H.; Prenger, J.J.; Rouzan-Wheeldon, D.T.; Levine, H.G.	A Comparison of Soil Moisture Sensors for Space Flight Applications	2005	Applied Engineering in Agriculture	21	211–216
Steinberg, S.L.; Kluitenberg, G.J.; Jones, S.B.; Daidzic, N.E.; Reddi, L.N.; Xiao, M.; Tuller, M.; Newman, R.M.; Or, D.; Alexander, J.I.D.	Physical and Hydraulic Properties of Baked Ceramic Aggregates Used for Plant Growth Medium	2005	Journal of the American Society for Horticultural Science	130	767–774

Elke Rabbow, Nevena Stojicic, David Walrafen, Christa Baumstark-Khan, Petra Rettberg, Dirk Schulze-Varnholt, Markus Franz, Günther Reitz	The SOS-LUX-TOXICITY-Test on the International Space Station	2006	Research in Microbiology	157	30–36
R. M. Wheeler, C. L. Mackowiak, G. W. Stutte, N. C. Yorio, L. M. Ruffe, J. C. Sager, R. P. Prince, W. M. Knott	Crop productivities and radiation use efficiencies for bioregenerative life support	2008	Advances in Space Research	41	706–713
Wheeler, Raymond M.; Stutte, Gar W.; Mackowiak, Cheryl L.; Yorio, Neil C.; Sager, John C.; Knott, William M.	Gas exchange rates of potato stands for bioregenerative life support	2008	Advances in Space Research	41	798–806
Kitaya, Y.; Hirai, H.	Effects of lighting and air movement on temperatures in reproductive organs of plants in a closed plant growth facility	2008	Advances in Space Research	41	763–767
Fu, Yuming; Guo, Rong; Liu, Hong	Dynamic simulation of Spirulina platensis growth and CO2 and O2 mass balance in BLSS	2009	Advances in Space Research	44	999-1007
Yeh, Naichia; Chung, Jen-Ping	High-brightness LEDs—Energy efficient lighting sources and their potential in indoor plant cultivation	2009	Renewable and Sustainable Energy Reviews	13	2175–2180

Lindsey K. Tuominen, Lanfang H. Levine, Mary E. Musgrave	Plant Secondary Metabolism in Altered Gravity	2009	Methods in Molecular Biology	547	373-386
Polyakov, Yuriy S.; Musaev, Ibrahim; Polyakov, Sergey V.	Closed bioregenerative life support systems: Applicability to hot deserts	2010	Advances in Space Research	46	775-786
Fjällman, T; Hall, JC	Design of a closed bioreactor for cultivation of higher plants in space	2010	Journal of Biomechanical Engineering	132	44504
Federico Maggi, Céline Pallud	Space agriculture in micro- and hypo-gravity: A comparative study of soil hydraulics and biogeochemistry in a cropping unit on Earth, Mars, the Moon and the space station	2010	Planetary and Space Science	58	1996–2007
Karen Olsson-Francis, Rosa de la Torre, Martin C. Towner, Charles S. Cockell	Survival of akinetes (cyanobacteria resting-state cells) in low Earth orbit and simulated extraterrestrial conditions	2010	Astrobiology	10	349-357
Schettini, E.	Biodegradability of greenhouse films	2011	Acta Horticulturae	891	785–790

Zongjie Hao, Dunhai Li, Yanhui Li, Zhicong Wang, Yuan Xiao, Gaohong Wang, Yongding Liu, Chunxiang Hu, Qifang Liu	Nostoc sphaeroides Kützing, an excellent candidate producer for CELSS	2011	Advances in Space Research	48	1565–1571
De Micco, Veronica; Buonomo, Roberta; Paradiso, Roberta; De Pascale, Stefania; Aronne, Giovanna	Soybean cultivar selection for Bioregenerative Life Support Systems (BLSS) – Theoretical selection	2012	Advances in Space Research	49	1415-1421
Bamsey, Matthew; Graham, Thomas; Thompson, Cody; Berinstain, Alain; Scott, Alan; Dixon, Michael	Ion-Specific Nutrient Management in Closed Systems: The Necessity for Ion-Selective Sensors	2012	Sensors	12	13349-13392
Cooper, Maya R.; Catauro, Patricia; Perchonok, Michele	Development and evaluation of bioregenerative menus for Mars habitat missions	2012	Acta Astronautica	81	555-562
De Micco, Veronica; Arena, Carmen; Di Fino, Luca; Narici, Livio	Soybean root growth and anatomy in microgravity	2012	Plant Biology	14 Suppl 1	50-58
Perchonok, Michele H.; Cooper, Maya R.; Catauro, Patricia M.	Mission to Mars: Food production and processing for the final frontier	2012	Annual Review of Food Science and Technology	3	311–330

Susan Fairburn, Barbara Imhof, Susmita Mohanty	Growing architecture: Investigating living systems in space design	2012	Acta Astronautica	81	428-439
Tiwari, Akhilesh; Fontaine, Jean-Pierre; Kondjoyan, Alain; Gros, Jean-Bernard; Vial, Christophe; Dussap, Claude-Gilles	Investigation of Interfacial Phenomena During Condensation of Humid Air on a Horizontal Substrate	2013	Oil & Gas Science and Technology - Revue de l IFP	68	959-973
Hu, Dawei; Zhang, Houkai; Zhou, Rui; Li, Ming; Sun, Yi	Controller development of photo bioreactor for closed-loop regulation of O2 production based on ANN model reference control and computer simulation	2013	Acta Astronautica	83	232-238
LeYuan Li, ZhiRuo Zhao, Hong Liu	Feasibility of feeding yellow mealworm (Tenebrio molitor L.) in bioregenerative life support systems as a source of animal protein for humans	2013	Acta Astronautica	92	103-109
Arena, C.; De Micco, V.; Aronne, G.; Pugliese, M.; De Santo, A. Virzo; De Maio, A.	Response of Phaseolus vulgaris L. plants to low-let ionizing radiation: Growth and oxidative stress	2013	Acta Astronautica	91	107–114
Elizabeth Kordyum; Karl H. Hasenstein	Plant Cell Gravisensitivity and Adaptation to Altered Gravity in Microgravity	2013	Plant Biology	15	dec-17

Paradiso, R.; De Micco, V.; Buonomo, R.; Aronne, G.; Barbieri, G.; De Pascale, S.	Soilless cultivation of soybean for Bioregenerative Life-Support Systems: a literature review and the experience of the MELiSSA Project	2014	Plant Biology	16(S1)	69-78
Dong, Chen; Hu, Dawei; Fu, Yuming; Wang, Minjuan; Liu, Hong	Analysis and optimization of the effect of light and nutrient solution on wheat growth and development using an inverse system model strategy	2014	Computers and Electronics in Agriculture	109	221–231
Shuangsheng Guo, Weidang Ai, Jinxue Fei, Guoxin Xu, Gu Zeng, Yunze Shen	Study on the kinetic characteristics of trace harmful gases for a two-person-30-day integrated CELSS test	2014	Environmental Science and Pollution Research	22	7020-7024
Roberta Paradiso, Roberta Buonomo, Mike A. Dixon, Giancarlo Barbieri, Stefania De Pascale	Effect of light intensity on microgravity-grown lettuce: morphological and quality aspects	2014	Acta Horticulturae	1037	931-938
Hu, Dawei; Li, Liang; Li, Yanchao; Li, Ming; Zhang, Houkai; Zhao, Ming	Gas equilibrium regulation by closed-loop photo bioreactor built on system dynamics, fuzzy inference system and computer simulation	2014	Computers and Electronics in Agriculture	103	114–121
Xu, Dongqian; Guo, Shuangsheng; Liu, Min	Effects of long-term simulated microgravity on tomato seedlings	2014	Canadian Journal of Plant Science	94	273–280

Ruyters, G.; Braun, M.	Gravitropism research in space: The ESA project on the ISS	2014	Microgravity Science and Technology	26	263–269
Kiss, John Z.; Aanes, Gjert; Schiefloe, Mona; Coelho, Liz H. F.; Millar, Katherine D. L.; Edelmann, Richard E.	Changes in operational procedures to improve spaceflight experiments in plant biology in the European Modular Cultivation System	2014	Advances in Space Research	53	818–827
L. Poulet, G.D. Massa, R.C. Morrow, C.M. Bourget, R.M. Wheeler, C.A. Mitchell	Significant reduction in energy for plant-growth lighting in space using targeted LED lighting and spectral manipulation	2014	Life Sciences in Space Research	2	43–53
G.W. Stutte	Optimization of the Veggie plant growth system for future space missions	2015	Open Agriculture Journal	8	
Hemant L. Gohil; Melanie J. Correll; Thomas Sinclair	Characterization of Oxygen Production, Photosynthesis, and Growth of Lemna Minor for a CELSS	2015	Life Sciences in Space Research	5	46-53
Leyuan Li, Michael Stasiak, Liang Li, Beizhen Xie, Yuming Fu, Danuta Gidzinski, Mike Dixon, Hong Liu	Rearing Tenebrio molitor in BLSS: Dietary fiber affects larval growth, development, and respiration characteristics	2016	Acta Astronautica	118	130-136

Li, Bowei; Dong, Chen; Chu, Zhengpei; Zhang, Weizhe; Wang, Minjuan; Liu, Hong; Xie, Beizhen	Synthesis, characterization and application of ion exchange resin as a slow-release fertilizer for wheat cultivation in space	2016	Acta Astronautica	127	579–586
L. Poulet, J.-P. Fontaine, C.-G. Dussap	Plant’s response to space environment: a comprehensive review including mechanistic modelling for future space gardeners	2016	Botany Letters	163	337-347
Thomas Graham; Raymond Wheeler	Nutrient solutions for lettuce grown in hydroponic systems on Earth and in space	2016	Life Sciences in Space Research	11	47-54
Ellen Polinski, Oliver Schueler, Lars Krause, Monika A. Wimmer, Ruth Hemmersbach, Heiner E. Goldbach	2-D clinorotation alters the uptake of some nutrients in Arabidopsis thaliana	2017	Journal of Plant Physiology	212	54–57
Chen Dong, Zhengpei Chu, Minjuan Wang, Youcai Qin, Zhihao Yi, Hong Liu, Yuming Fu	Influence of nitrogen source and concentrations on wheat growth and production inside 'Lunar Palace-1'	2018	Acta Astronautica	144	371-379
D.T. Peterside, J.E. Palaia, A.C. Schuerger, R.A. Bucklin, M.J. Correll	Testing of greenhouse cladding materials for space environments, Part 2: Laminates	2018	Applied Engineering in Agriculture	34	575-580

N. Fulget, L. Poughon, J. Richalet, C. Lasseur	Optimization of the design of MELiSSA gas-liquid transfer compartments by the development of multiphase numerical models	2018	Life Sciences in Space Research	19	36-48
Yang, Limin; Li, Huankai; Liu, Tonggui; Zhong, Yuqing; Ji, Chengcheng; Lu, Qian; Fan, Liangliang; Li, Jun; Leng, Lijian; Li, Kun; Zhou, Wenguang	Microalgae biotechnology as an attempt for bioregenerative life support systems: problems and prospects	2019	Journal of Chemical Technology and Biotechnology	94	3039-3048
C. Arena, E. Vitale, B. Hay Mele, P. R. Cataletto, M. Turano, P. Simoniello, V. De Micco	Suitability of Solanum lycopersicum L. 'Microtom' for growth in Bioregenerative Life Support Systems	2019	Plant Biology	21	615–626
Minjuan Wang, Chen Dong, Wanlin Gao	Study on the wheat growth, photosynthesis and yield in BLSS	2019	Acta Astronautica	157	463-471
Matthias C. Rillig, Janis Antonovics	Microbial evolutionary rescue and the time scale for recovery of ecosystems after disturbance	2019	ISME Journal	13	1230–1234
Marzioli, Paolo; Gugliermetti, Luca; Santoni, Fabio; Delfini, Andrea	CultCube: Experiments in autonomous in-orbit cultivation on-board a 12-Units CubeSat platform	2020	Life Sciences in Space Research	25	42–52

Chen, Yanwu; Xu, Chong; Zhong, Chongfa; Lyu, Zhitang; Liu, Junlian; Chen, Zhanghuang; Dun, Huanhuan; Xin, Bingmu; Xie, Qiong	Study on biodegradable packaging material and its impact on waste management in bioregenerative life support systems	2020	Waste Management	102	846-856
Hu, Dawei; Zhang, Houkai; Li, Leyuan; Zhou, Rui; Sun, Yi	Biomass conversion and energy utilization efficiencies of a soil-like substrate bioreactor for solid waste treatment in bioregenerative life support systems	2020	Acta Astronautica	173	137-144
Sofo, Adriano	UV-C rays to simulate the exposition of photosynthetic organisms to solar radiation in space environments	2020	International Journal of Plant Biology	11	19-25
Jianxing Wang, Yuansong Wei	Recovery of monovalent mineral salts from urine in controlled ecological life support system by nanofiltration: Feasibility study	2020	Desalination	479	114344
Conrad Zeidler; Gerrit Woeckner; Johannes Schoening; Vincent Vrakking; Paul Zabel; Markus Dorn; Daniel Schubert; Birgit Steckelberg	EDEN ISS - A greenhouse model for future food production in space	2020	Acta Astronautica	176	341-353
Cycil, Leena M.; Hausrath, Elisabeth M.; Ming, Douglas W.; Adcock, Christopher T.; Raymond, James; Remias, Daniel; Ruemmele, Warren P.	Investigating the Growth of Algae Under Low Atmospheric Pressures for Potential Food and Oxygen Production on Mars	2021	Frontiers in Microbiology	12	733244

Kordyum, Elizabeth; Chapman, David; Brykova, Vasyl	Effects of Microgravity on Plant Morphogenesis and Adaptation Mechanisms	2021	Life Sciences in Space Research	29	56-63
Ciurans, Carles; Guerrero, Josep M.; Martinez-Mongue, Ivan; Dussap, Claude G.; de Mas, Igor Marin; Godia, Francesc	Enhancing control systems of higher plant culture chambers via multilevel structural mechanistic modelling	2022	Frontiers in Plant Science	13	970410
Duri, Luigi Giuseppe; Caporale, Antonio Giandonato; Rouphael, Youssef; Vingiani, Simona; Palladino, Mario; De Pascale, Stefania; Adamo, Paola	The Potential for Lunar and Martian Regolith Simulants to Sustain Plant Growth: A Multidisciplinary Overview	2022	Frontiers in Astronomy and Space Sciences	8	747821
Anne M. Visscher, Hugh W. Pritchard, Gianluca Neri, Daniel Ballesteros	How do we transport plant species with desiccation-sensitive germplasm in space?	2022	Life Sciences in Space Research	36	135-137
Xiaofeng Liu, Min Chen, Zuliang Bian, Chung-Chu Liu	Microalgae-based bioregenerative life support systems: progress and perspectives	2022	Frontiers in Microbiology	13	968875
Sakhare, Shreyash A.; Pendkar, Sourabh M.; Kanu, Nand Jee; Gupta, Eva; Vates, Umesh Kumar; Singh, Gyanendra Kumar; Verma, Girish C.	Design suggestions on modified self-sustainable space toilet	2022	SN Applied Sciences	4	13

Kovalev, V. S.; Grandl, W.; Manukovsky, N. S.; Tikhomirov, A. A.; Boeck, C.	Modeling a lunar base mushroom farm	2022	Life Sciences in Space Research	33	1–6
Alicia Villacampa; Malgorzata Ciska; Aranzazu Manzano; Joshua P. Vandenbrink; John Z. Kiss; Raul Herranz; F. Javier Medina	Influence of gravity on cell wall-related genes in Arabidopsis thaliana seedlings grown in space	2022	International Journal of Molecular Sciences	23	4258
Sukhinov, Daniil V.; Gotovtsev, Pavel M.; Sergeeva, Yana E.	Phototrophic microorganisms in bioregenerative life support systems for long-term crewed expeditions: Prospects and challenges	2023	Acta Astronautica	211	518-538
Anna Jurga, Anna Pacak, Demis Pandelidis, Bartosz Kazmierczak	Condensate as a water source in terrestrial and extra-terrestrial conditions	2023	Water Resources and Industry	29	100196
Benjamin Trotter, Kathrin A. Otte, Kathrin Schoppmann, Ruth Hemmersbach, Thomas Froehlich, Georg J. Arnold, Christian Laforsch	Long-term space exposure of Daphnia magna – first insights into alterations of molecular pathways	2023	Science Advances	9	eadf1246
Laura Alemany, Enrique Peiro, Carolina Arnau, David Garcia, Laurent Poughon, Jean-Francois Cornet, Claude-Gilles Dussap, Olivier Gerbi, Brigitte Lamaze	Nutrient recycling from inedible biomass and its potential integration into a MELiSSA loop: new insights and perspectives	2023	npj Microgravity	9	11

Kuzma, Joanna; Poulet, Lucie; Fontaine, Jean-Pierre; Dussap, Claude-Gilles	Modelling physical processes in higher plants using leaf replicas for space applications	2023	Comptes Rendus Mécanique	351	97–113
Wheeler, R. M.; Wehkamp, C. A.; Stasiak, M. A.; Dixon, M. A.; Rygalov, V. Y.	Challenges for Consideration When Designing Space Agriculture Systems for Human Life Support	2023	Frontiers in Astronomy and Space Sciences	10	1192437
Leone Ermes Romano, Jack J. W. A. van Loon, Luigi Gennaro Izzo, Maurizio Iovane, Giovanna Aronne	Effects of altered gravity on growth and morphology in Wolffia globosa: implications for bioregenerative life support systems and space-based agriculture	2024	Scientific Reports	14	410
P. Joris, E. Lombard, A. Paillet, G. Navarro, S.E. Guillouet, N. Gorret	Recycling potential of Cupriavidus necator for life support in space: Production of SCPs from volatile fatty acid and urea mixture	2024	Journal of Biotechnology	396	18–27
Ohya, H.; Oshima, T.; Nitta, K.	Survey of CELSS Concepts and Preliminary Research in Japan	1985	NASA Technical Report	4	271-277
Rummel, J D; Volk, T	A Modular BLSS Simulation Model	1988	NASA Technical Report	7	59-67

PETERSEN, GR	Microbial Bioreactors for Waste Treatment and Resource Recovery	1989	Biological Life Support Technologies		127-135
Gonzales, AA; Schuerger, AC; Barford, C; Mitchell, R	Microbial aerosols in a closed life support system	1993	Advances in Space Research	12	119–127
V. Blüm, M. Andriske, C. Ludwig, U. Paassen, D. Voeste	The closed equilibrated biological aquatic system (C.E.B.A.S.) as a possible model for aquatic food production in a bioregenerative life support system	1994	Acta Astronautica	32	131-137
V. Bluem; M. Andriske; F. Paris; D. Voeste	The Dynamic Aquatic System (AQUARACK): A model for closed life support system research	1994	Acta Astronautica	32	131-138
Gorgolewski, S	Resource Recycling in Closed Life Support Systems	1995	Acta Astronautica	35	565-573
Victorov, AN; Ilyin, VK; Syniak, JE	The problems of microbial safety in regenerative life support systems	1995	Acta Astronautica	35	513–576

Lasseur, C.; Verstraete, W.; Gros, JB.; Dubertret, G.; Rogalla, F.	MELISSA: A Potential Experiment for a Precursor Mission to the Moon	1996	Advances in Space Research	18	111-117
BLACKWELL, CC	Performance Robustness of Biological Life Support Systems	1996	Advances in Space Research	18	167-170
Kordyum, E.L.	PLANT REPRODUCTION SYSTEMS IN MICROGRAVITY: EXPERIMENTAL DATA AND HYPOTHESES	1998	Advances in Space Research	21	1111-1120
S.I. Bartsev, V.A. Okhonin	SELF-RESTORATION OF BIOCOMPONENTS AS A MEAN TO ENHANCE BIOLOGICAL LIFE SUPPORT SYSTEMS RELIABILITY	1999	Advances in Space Research	24	393-396
John Allen, Mark Nelson	Biospherics and Biosphere 2, mission one (1991–1993)	1999	Ecological Engineering	13	15–29
Paille, C; Albiol, J; Curwy, R; Lasseur, C; Godia, F	FEMME: a precursor experiment for the evaluation of bioregenerative life support systems	2000	Planetary and Space Science	48	515–521

Bluem, V.; Paris, F.	Aquatic modules for bioregenerative life support systems based on closed aquaria and fish breeding	2001	Acta Astronautica	48	287-297
L.S. Tirranen	Formation of Higher Plant Component Microbial Community in Closed Ecological System	2001	Acta Astronautica	49	47–52
Gòdia, F; Albiol, J; Montesinos, JL; Pérez, J; Creus, N; Cabello, F; Mengual, X; Montras, A; Lasseur, C	Preliminary results of the MELISSA continuous culture system and its integration within bioregenerative life support systems	2002	Advances in Space Research	31	1995-2004
Sakano, Y; Pickering, KD; Strom, PF; Kerkhof, LJ	Effect of environmental factors on nitrogen removal from wastewater by an anaerobic ammonium oxidation biofilm reactor	2002	Applied and Environmental Microbiology	68	4509–4516
D. Voeste, L.H. Levine, H.G. Levine, V. Blüm	Pigment Composition and Concentrations within the Plant (Ceratophyllum demersum L.) Component of the STS-89 C.E.B.A.S. Mini-Module Spaceflight Experiment	2002	Advances in Space Research	31	211-214
Bartsev, S.I.; Mezhevikin, V.V.; Okhonin, V.A.	Systematic Approach to Life Support System Analyses and Integration	2003	Advances in Space Research	31	1823-1832

V. Blüm	Aquatic Modules for Bioregenerative Life Support Systems: Developmental Aspects Based on the Space Flight Results of the C.E.B.A.S. Mini-Module	2003	Advances in Space Research	31	1683-1691
O. Monje; G.W. Stutte; G.D. Goins; D.M. Porterfield; G.E. Bingham	Farming in Space: Environmental and Biophysical Concerns	2003	Advances in Space Research	31	151-167
Gòdia, F; Albiol, J; Pérez, J; Creus, N; Cabello, F; Montràs, A; Masot, A; Lasseur, C	The MELISSA pilot plant facility as an integration test-bed for advanced life support systems	2004	Advances in Space Research	34	1483–1493
Ishikawa, Y.; Yoshida, H.; Kinoshita, M.; Murakami, A.; Sugiura, K.	Examination of a smallest CELSS (microcosm) through an individual-based model simulation	2004	Advances in Space Research	34	1517–1527
Farges, Berangere; Poughon, Laurent; Creuly, Catherine; Cornet, Jean-Francois; Dussap, Claude-Gilles; Lasseur, Christophe	Dynamic Aspects and Controllability of the MELiSSA Project: A Bioregenerative System to Provide Life Support in Space	2008	Applied Biochemistry and Biotechnology	151	686–699
Nelson, Mark; Pechurkin, Nickolay S.; Allen, John P.; Somova, Lydia A.; Gitelson, Josef I.	Closed Ecological Systems, Space Life Support and Biospherics	2009	Handbook of Environmental Engineering	10	517-565

Gonzales, John M. Jr.	Aquaculture in bio-regenerative life support systems (BLSS): Considerations	2009	Advances in Space Research	43	1250–1255
Kai Zhang; Hyeok Choi; Dionysios D. Dionysiou; Daniel B. Oerther	Assessment of Microbial Communities in a Membrane Bioreactor Treating Spacecraft Wastewater Simulant	2009	Journal of Environmental Engineering	135	836-845
Maggi, Federico; Pallud, Celine	Martian base agriculture: The effect of low gravity on water flow, nutrient cycles, and microbial biomass dynamics	2010	Advances in Space Research	46	1257–1265
Mark Nelson, Hinrich L. Bohn	Soil-Based Biofiltration for Air Purification: Potentials for Environmental and Space Life Support Application	2011	Journal of Environmental Protection	2	1084-1094
Hu, Dawei; Zhou, Rui; Sun, Yi; Tong, Ling; Li, Ming; Zhang, Houkai	Construction of closed integrative system for gases robust stabilization employing microalgae peculiarity and computer experiment	2012	Ecological Engineering	44	78-87
Kordyum, E. L.	Biology of plants in space: scientific results and problems	2013	Kosmichna Nauka i Tekhnologiya	19	65-77

Xue Liang, Yuming Fu, Ling Tong, Hong Liu	Microbial shifts of the silkworm larval gut in response to lettuce leaf feeding	2014	Applied Microbiology and Biotechnology	98	3769–3776
Steinberg, L.M.; Kronyak, R.E.; House, C.H.	Coupling of anaerobic waste treatment to produce protein- and lipid-rich bacterial biomass	2017	Life Sciences in Space Research	15	32–42
König, B.; Dünne, M.; Slenzka, K.	Integration of aquatic animals into bioregenerative life support systems – A review	2018	Acta Astronautica	146	409-417
Haeder, Donat-P.	On the Way to Mars—Flagellated Algae in Bioregenerative Life Support Systems Under Microgravity Conditions	2020	Frontiers in Plant Science	10	1621
Garegnani, Marco; Nardi, Laura; Calvitti, Sandro.	Lepidoptera: potential pests of crops in space	2020	Acta Astronautica	176	184-193
Hu, Dawei; Li, Ming; Zhou, Rui; Sun, Yi	Operational mode of a gas-tight photo-bioreactor coupling nitrifying bacteria and microalgae for oxygen regeneration	2020	Ecological Engineering	158	106090

Poughon, Laurent; Creuly, Catherine; Godia, Francesc; Leys, Natalie; Dussap, Claude-Gilles	Mathematical modeling of the MELiSSA closed life support system: Optimizing mass flows and greenhouse area	2020	Life Sciences in Space Research	24	juli-17
Kovalev, VS; Manukovsky, NS; Tikhomirov, AA; Kolmakova, AA	Effect of microbial inoculation on wheat growth and photosynthetic productivity under controlled environment	2020	Acta Astronautica	170	617-623
Kovalev, V. S.; Manukovsky, N. S.; Tikhomirov, A. A.	Bioregenerative life support space diet and nutrition requirements: still seeking accord	2020	Life Sciences in Space Research	27	99–104
Jianlou Yang, Zikai Hao, Lantao Zhang, Yuming Fu, Hong Liu	A newly developed plant-microbial bioreactor for nitrogen recovery from urine	2020	Acta Astronautica	176	241-249
Zheng, Libing; Zhang, Chun; Gao, Rui; Zhang, Liangchang; Ai, Weidang; Ulbricht, Mathias; Wei, Yuansong	Design and operation of a ceramic membrane bioreactor for wastewater treatment in a bioregenerative life support system	2021	Science of the Total Environment	770	144597
Lemos, M.F.L.	Environmental monitoring: Benefits of using multiple biotests	2021	Ecotoxicology and Environmental Safety	222	112490

Caporale, Antonio G.; Vingiani, Simona; Palladino, Mario; El-Nakhel, Christophe; Duri, Luigi G.; Pannico, Antonio; Rouphael, Youssef; De Pascale, Stefania; Adamo, Paola	Biochar application in space cultivation: A review of plant-soil-microbe interactions and terrestrial analogs	2022	Frontiers in Astronomy and Space Sciences	9	873725
Morozov, Yegor; Kudenko, Yurii; Trifonov, Sergey; Tikhomirov, Alexander	Experimental Investigation of Nitrogen Cycle Components in a Bioregenerative Life Support System	2022	Life Sciences in Space Research	35	35–44
Thomas Graham, Raymond Wheeler	Feeding astronauts on long-duration space missions: perspectives from terrestrial animal production systems	2023	Life Sciences in Space Research	38	37-48
Domokos-Szabolcsy, Eva; Alshaal, Tarek; Koroknai, Judit; Kovacs, Szilvia; Toth, Csaba; Csillery, Gabor; Jokai, Zsuzsa; Matkovits, Anna; Makleit, Peter; Veres, Szilvia	Phytochemical evaluation of the fruits and green biomass of determinate-type sweet pepper (Capsicum annuum L. fasciculatum) grown in terrestrial bioregenerative life-support research facilities	2023	Journal of Plant Interactions	18	2268118
Yadav, Anu; Monsieurs, Pieter; Misztak, Agnieszka; Waleron, Krzysztof; Leys, Natalie; Cuypers, Ann; Janssen, Paul J.	Biological wastewater treatment for nutrient recovery in a closed loop life support system for space	2023	Science of The Total Environment	899	165667
Matula, Emily E.; Nabity, James A.; McKnight, Diane M.	Nitrification activity in hydroponic root zone nutrient delivery systems for spacecraft plant habitats	2023	npj Microgravity	9	26

Curry, Aaron B.; Spern, Cory J.; Khodadad, Christina L. M.; Hummerick, Mary E.; Spencer, LaShelle E.; Torres, Jacob; Finn, J. Riley; Gooden, Jennifer L.; Monje, Oscar	Microbiological assessment of romaine lettuce grown in Veggie on the International Space Station	2023	npj Microgravity	9	30
Matula, Emily E.; Nabity, James A.	Urea hydrolysis in controlled release fertilizer for spacecraft plant production systems	2023	Acta Astronautica	208	130–139
Romano, Donato; Di Giovanni, Adriano; Pucciariello, Chiara; Stefanini, Cesare	Turning earthworms into moonworms: Earthworms colonization of lunar regolith as a bioengineering approach supporting future crop growth in space	2023	Heliyon	9	e14683
Ryan Keller, Karthik Goli, William Porter, Aly Alrabaa, Jeffrey A. Jones	Cyanobacteria and Algal-Based Biological Life Support System (BLSS) and Planetary Surface Atmospheric Revitalizing Bioreactor Brief Concept Review	2023	Life	13	816
Giannetta, B.; Caporale, A.G.; Olivera de Souza, D.; Adamo, P.; Zaccone, C.	Evidence of Potential Organo-Mineral Interactions during the First Stage of Mars Terraforming	2023	Soil Systems	7	92
Sole, Ricard; Maull, Victor; Amor, Daniel R.; Mauri, Jordi Pla; Nuria, Conde-Pueyo	Synthetic Ecosystems: From the Test Tube to the Biosphere	2024	ACS Synthetic Biology	13	3812–3826
