# Supplementary material for: Insects in bioregenerative life support systems: unlocking their role in space sustainability
Source: Front Physiol. 2025 Sep 10;16:1621099. doi: 10.3389/fphys.2025.1621099 (PMC12457449; doi:10.3389/fphys.2025.1621099)
Supplement: Supplementary file 2 [file Table2.pdf]

Table S2. Nutritional composition of four edible insect species approved for consumption in the European Union. Values are presented on a dry matter (DM) basis and include crude protein, fat content, essential amino acid concentrations (g per 100 g protein), major fatty acids (percentage of total fat), and selected minerals and vitamin B_{12}. Data are primarily compiled from Oonincx and Finke (2021), with contextual support from van Huis et al. (2021). Reported values represent typical ranges but may vary depending on species, developmental stage, rearing substrate, processing methods, and environmental conditions.

Species	Crude Protein (% DM)	Fat (% DM)	Lysine (g/100g protein)	Methionine (g/100g protein)	Tryptophan (g/100g protein)	Linoleic acid (omega-6, % fat)	Alpha-linolenic acid (omega-3, % fat)	Oleic acid (omega-9, % fat)	Iron (mg/100g DM)	Zinc (mg/100g DM)	Calcium (mg/100g DM)	Vitamin B12 (µg/100g DM)
Acheta domesticus (house cricket)	69.1	15.4	6.0	1.8	1.1	38.0	2.5	25.0	9.5	20.3	130.0	2.2
Tenebrio molitor (yellow mealworm)	53.4	33.9	6.2	1.5	1.2	42.3	1.3	30.4	5.8	10.2	55.0	2.1
Alphitobius diaperinus (lesser mealworm),	55.0	29.9	6.1	1.6	1.1	40.1	1.8	28.9	4.7	9.9	60.0	1.8
Locusta migratoria (migratory locust)	70.1	13.4	6.3	1.7	1.2	35.0	2.1	23.5	6.3	14.5	90.0	1.7
